# Shengmai Injection as adjunctive therapy in sepsis and septic shock: a retrospective cohort study

**DOI:** 10.3389/fcimb.2026.1870034

**Published:** 2026-07-30

**Authors:** Ya Li, Xiaohui Chen, Qingmin Li, Lulu Wu, Liyuan Yu, Weihang Peng, Yongshang Luo, Peiying Huang, Ye Ye, Bojun Chen, Qiuping Huang, Li Chen

**Affiliations:** 1 The Second Clinical Medical College, Guangzhou University of Chinese Medicine, Guangzhou, China; 2 Cardiology Department, The First People’s Hospital of Chenzhou, Chenzhou, China; 3 School of Traditional Chinese Medicine, Guangdong Pharmaceutical University, Guangzhou, China; 4 Acupuncture and Rehabilitation Department, Guangdong Provincial Second Hospital of Traditional Chinese Medicine, Guangzhou, China; 5 Emergency Department,The Second Affiliated Hospital of Guangzhou University of Chinese Medicine, Guangzhou, China

**Keywords:** Shengmai injection, Sepsis, Septic shock, Adjunctive therapy, Retrospective study

## Abstract

**Background:**

Sepsis and septic shock constitute critical global health burdens associated with substantial mortality. Shengmai Injection (SMI), a classic traditional Chinese medicine formulation endowed with anti-inflammatory and immunomodulatory properties, has demonstrated efficacy as an adjunctive therapy in critical illness. Nevertheless, high-level clinical evidence supporting its routine application remains limited. This work sought to systematically explore the therapeutic efficacy of adjunctive SMI intervention in patients with sepsis and septic shock.

**Materials and methods:**

Mass spectrometry was employed to identify chemical constituents of SMI. This multicenter retrospective cohort study enrolled patients meeting Sepsis 3.0 diagnostic criteria for sepsis or septic shock at the Guangdong Provincial Hospital of Traditional Chinese Medicine from February 2016 to June 2024. After 1:1 propensity score matching (PSM) to balance baseline confounders, clinical outcomes were compared between the SMI and control groups. The primary endpoint was 28-day mortality. Secondary outcomes included hospital and intensive care unit (ICU) stay, ventilation and fever duration, and changes in SOFA score, APACHE II scores, procalcitonin (PCT), lactate, and blood cell counts at days 3–7 post-treatment. A subgroup analysis was further conducted among patients with septic shock.

**Results:**

A total of 130 components were identified in SMI. The retrospective study involving 436 enrolled patients (136 in the septic shock subgroup) showed that SMI shortened ICU stay, ventilation duration, and fever duration, and reduced post-treatment SOFA, APACHE II scores, PCT, platelet count, and lactate levels. Cox regression analysis revealed that post-treatment SOFA score (HR = 1.133), PCT (HR = 1.024), lactate (HR = 1.432), and SMI course (HR = 0.830) were independent predictors of 28-day mortality. Subgroup analysis of 72 patients with septic shock demonstrated that SMI reduced invasive ventilation duration, improved post-treatment SOFA and APACHE II scores, and decreased platelet and lactate levels. However, SMI did not significantly reduce 28-day mortality in patients with sepsis or septic shock.

**Conclusion:**

Adjunctive SMI therapy was associated with certain beneficial physiological effects, including improvements in inflammatory and metabolic markers and organ function, without conferring a significant survival benefit regarding 28-day mortality. Post-treatment PCT and lactate levels serve as powerful prognostic biomarkers for disease progression. Further prospective randomized controlled trials (RCTs) are required to corroborate these observations and consolidate clinical evidence.

## 1 Introduction

Sepsis is defined as a life-threatening clinical syndrome characterized by organ dysfunction resulting from a dysregulated host response to infection (Singer et al., 2016). Recent evidence indicates that although incidence and mortality rates of sepsis have decreased in certain countries, the disease remains a global public health issue due to its escalating mortality burden and persistent long-term complications (Singer et al., 2026). Emerging studies have confirmed survivors are highly susceptible to post-sepsis syndrome, a debilitating complication manifested as physical dysfunction, neurocognitive deficits, and mood disorders. These adverse long-term sequelae may persist for up to 5 years (Palakshappa and Taylor, 2025). The epidemiological data further demonstrated that sepsis causes 11 million deaths annually, accounting for 19.7% of global mortalities (Rudd et al., 2020). As the most severe clinical form of sepsis, septic shock is characterized by severe disturbances in circulatory, cellular, and metabolic homeostasis, with a mortality rate as high as about 38.5% even in developed countries (Bauer et al., 2020).

Current management of sepsis and septic shock emphasizes rapid diagnosis, infection control, eradication, fluid resuscitation, and organ support (Singer et al., 2026). Advances in mechanistic understanding have also established immunotherapy as a key therapeutic strategy. While specific immunomodulatory treatments for sepsis are not yet available, modulating the host immune response may improve long-term outcomes (Marques et al., 2023). However, challenges such as antibiotic resistance, inadequate fluid resuscitation, ventilator-induced lung injury, and the high heterogeneity of sepsis highlight the urgent need for novel adjuvant therapies to halt disease progression, reduce mortality, and improve prognosis (de la Fuente-Nunez et al., 2023; Ward et al., 2025). Notably, growing evidence suggests that Traditional Chinese Medicine (TCM) holds considerable therapeutic potential and promising clinical prospects in sepsis treatment.

Shengmai Injection (SMI), a patented Chinese herbal formula, is composed of *Panax ginseng* C.A.Mey. (Hong Shen), *Ophiopogon japonicus* (Linn. f.) Ker-Gawl (Mai Dong), and *Schisandra chinensis* Turcz. (Baill.) (Wu Weizi). Accumulating evidence has validated its multi-organ-protective properties (Wu et al., 2025). From a TCM perspective, SMI exerts remarkable therapeutic actions through replenishing qi, fluid secretion, and astringing yin to arrest pathological diaphoresis. With long-standing clinical application history, SMI has been widely adopted in the intervention of sepsis spectrum disorders, such as sepsis-induced myocardial injury, hepatic insufficiency, and septic shock. Mounting clinical investigations have demonstrated that SMI significantly improved multiple clinical prognostic indicators, including lowered Sequential Organ Failure Assessment (SOFA) and Chronic Health Evaluation (APACHE) II scores, and diminished serum concentrations of C-reactive protein (CRP), PCT, interleukin-6(IL-6), and lactate. Meanwhile, such intervention effectively shortened ICU hospitalization and reduced mortality in septic individuals (Chen et al., 2015b; Yang et al., 2021; Yao et al., 2021). Pharmacological evidence has substantiated that SMI contains a diverse array of bioactive constituents, including Ginsenoside Rb_1_, Ginsenoside Rg_1_, Ophiopogonin, Ophiopogon flavonoids, and Schisandrin. Functionally, these components possess robust anti-inflammatory and immunomodulatory capacities and exert protective actions on microcirculation. Such pharmacological activities collectively attenuate sepsis-elicited systemic inflammation and progressive tissue injury (Shi et al., 2025).

Although preliminary evidence has validated the favorable therapeutic effects of SMI against sepsis, existing studies are small−sample, single−center RCTs with low methodological quality. Several investigations lacked strict control of concurrent Chinese herbal use, employed limited outcome measures focused primarily on short−term physiological scores and inflammatory markers, and omitted both dose−response analyses and independent prognostic factor evaluations. These limitations collectively undermine the reliability and generalizability of existing evidence regarding SMI in sepsis management. Given such prominent research limitations, high-level clinical evidence is urgently needed to clarify the mechanistic therapeutic potential and translational applicability of SMI in sepsis management. Accordingly, the present retrospective cohort study was conducted to comprehensively evaluate the clinical efficacy of SMI in patients with sepsis and septic shock by comparing SMI-exposed and unexposed cohorts.

## 2 Methods

### 2.1 UPLC-MS analysis for SMI

Comprehensive phytochemical profiling of bioactive constituents within SMI was performed using means of ultra-high-performance liquid chromatography-tandem mass spectrometry (UPLC-MS/MS). Separation procedures were conducted on a Waters Acquity UPLC apparatus with a UPLC HSS T3 column (2.1 × 100 mm, 1.8 μm) at a constant temperature of 35 °C. The mobile phase consisted of acetonitrile (A) and 0.2% formic acid aqueous solution (B), with a flow rate of 0.3 mL min⁻¹ and injection volume of 5 μL. Mass spectrometric detection was conducted on a hybrid triple quadrupole-ion trap mass spectrometer coupled to the chromatographic system, operating simultaneously in positive and negative electrospray ionization modes. With collision energies adjusted to 25, 35, and 45 eV, full-scan mass spectra were obtained over an m/z range of 100-2000. Putative compound identification was performed by systematically comparing high-resolution precursor masses and characteristic tandem mass fragmentation spectra with a proprietary in-house reference library.

### 2.2 Retrospective cohort study

#### 2.2.1 Ethics

Ethical approval for this retrospective study was granted by the Institutional Review Board, which also exempted the requirement for writing informed consent. Due to the retrospective nature of the study, the use of fully anonymized patient data, and the absence of any commercial interests, the waiver complied entirely with national and institutional regulations (Chen et al., 2026; Peng et al., 2026; Tang et al., 2025).Moreover, the present investigation was conducted in strict conformity with the ethical principles outlined in the World Medical Association Declaration of Helsinki.

#### 2.2.2 Data sources

Consecutive patients diagnosed with sepsis or septic shock were enrolled in this retrospective study. All pertinent clinical datasets were retrospectively extracted from the centralized electronic medical record platform at Guangdong Provincial Hospital of Traditional Chinese Medicine, covering four distinct hospital campuses: Dade Road Headquarters, Guangzhou Higher Education Mega Center Campus, Fangcun Branch, and Ersha Island Branch. The study encompassed an observation period ranging from February 23, 2016, to June 30, 2024.

#### 2.2.3 Selection criteria

##### 2.2.3.1 Inclusion criteria

1. Aged ≥18 years.
2. Definitive diagnosis of sepsis or septic shock was confirmed in strict accordance with the Sepsis 3.0 criteria, with the diagnostic confirmation completed within 72 hours of study enrollment (Singer et al., 2016).
3. Post-diagnosis administration of SMI for ≥24 hours, and no exposure to any herbal medicinal preparations.
4. Availability of complete clinical basic characteristics and laboratory data at admission, including demographics, vital signs (heart rate, systolic and diastolic blood pressure, body temperature, respiratory rate, peripheral capillary oxygen saturation (SPO_2_)), primary infection source, underlying conditions, SOFA scores, APACHE II scores, anti-infective regimens, blood culture results, PCT levels, peripheral blood cell counts (white blood cells, platelets, lymphocytes, neutrophils, neutrophil-to-lymphocyte ratio (NLR)) and blood lactate concentration.
5. Comprehensive retrievable outcome data, including 28-day mortality, length of hospital and ICU stay, duration of non-invasive and invasive ventilation, duration of continuous renal replacement therapy (CRRT), fever duration, as well as SOFA scores, APACHE II scores, PCT, peripheral blood cell counts, and blood lactate concentration on post-treatment days 3-7.

##### 2.2.3.2 Exclusion criteria

1. Pregnant or lactating women, and patients with psychiatric disorders.
2. Severe comorbidities, including coagulation disorders, advanced malignant tumors, acquired immunodeficiency syndrome, post-cardiopulmonary resuscitation status, severe cardio-cerebrovascular diseases (e.g., acute coronary syndrome), and pre-existing organ dysfunction (e.g., chronic renal failure).
3. Organ transplantation or immunosuppressant use within the past 6 months.
4. Concurrent enrollment in other clinical trials.
5. Allergy to SMI.

#### 2.2.4 Treatment protocols

All patients enrolled in this study were administered standardized conventional Western therapeutic regimens in strict accordance with clinical guidelines governing the management of sepsis and septic shock (Singer et al., 2026). Beyond baseline routine treatment, individuals in the SMI group received supplementary intravenous infusion of SMI (10 mL per ampoule). The daily dosage was adjusted between 20 and 100 mL based on personalized disease severity and prescribing guidelines. All patients allocated to the intervention group were required to complete at least 24 hours of continuous SMI treatment. The SMI medication course was defined as the actual number of treatment days during which the patient received SMI infusion based on their ill condition and drug response. Notably, concomitant use of alternative traditional Chinese herbal medications was excluded across the entire study period.

#### 2.2.5 Outcome indicators

The primary outcome measure was defined as 28-day mortality. Secondary efficacy indicators included the length of hospital stay, length of ICU stay, duration of mechanical ventilator (encompassing both non-invasive and invasive ventilation), duration of CRRT, as well as SOFA scores, APACHE II scores, PCT, white blood cells, platelets, lymphocytes, neutrophils, NLR, and blood lactate level measured on days 3–7 of treatment.

#### 2.2.6 Statistical analysis

To alleviate intergroup confounding attributable to imbalanced patient proportions and divergent baseline profiles, PSM was performed using 1:1 optimal non-replacement matching (without a caliper), incorporating key baseline covariates for the sepsis and septic shock cohort. Analyses were conducted in R (v4.5.2) utilizing the MatchIt package. Optimal matching was determined by evaluating standardized mean difference (SMD), variance ratio, and empirical cumulative distribution function (eCDF) metrics, with optimal balance achieved when the eCDF approached 0 and the variance ratio converged to 1 (Huang et al., 2025). Specifically, an SMD less than 0.1 indicated an optimal balance, an SMD between 0.1 and 0.2 indicated a mild residual imbalance, and an SMD greater than 0.2 indicated a prominent, unacceptable imbalance (Austin, 2009; Zeng et al., 2024). Variables with mild residual imbalance or p<0.05 were entered into multivariate Cox regression to adjust for residual confounding and validate result reliability. Love plots were subsequently adopted to visualize covariate distribution changes before and after matching.

For inter-group comparisons, continuous variables were expressed as mean ± standard deviation (SD) for normally distributed data (Shapiro-Wilk test) or as median and interquartile range (IQR) for non-parametric data. Subsequently, the t-test or Mann–Whitney U test was used for comparative analysis of normally and non-normally distributed variables, respectively. Categorical variables were presented as frequencies and percentages, and intergroup disparities were evaluated using the chi-square test. Cox proportional hazards regression and linear regression models were subsequently used to assess the associations between explanatory variables and the primary endpoint, as well as significant secondary outcome metrics. Kaplan-Meier methodology was employed to generate survival probability curves, whereas the log-rank test was applied for subgroup comparative analysis to verify independent prognostic predictive significance (Yang et al., 2025). Covariates were selected based on statistical significance and clinical rationality. Specifically, the “SMI medication course” was included as a continuous covariate representing the total duration of drug exposure (as defined in Section 2.2.4), and medication dosages were also treated as a continuous covariate in subsequent analyses, given their highly concentration and the limited sample size. Statistical analyses were performed by using SPSS 25.0 and R software (version 4.5.2). A two-sided *p* < 0.05 was considered statistically significant. To evaluate the reliability and robustness of the analytical outcomes, sensitivity testing using Rosenbaum bounds(via the McNemar test, *Γ* = 1.0) following PSM, as well as a *post-hoc* power analysis and sample size calculation using GPower 3.1, were conducted. In light of septic shock being identified as a life-threatening severe subtype within the sepsis disease spectrum, stratified subgroup analyses were additionally performed to elucidate the therapeutic efficacy of SMI intervention. Importantly, the septic shock subgroup was subjected to the same rigorous statistical procedures and analytical frameworks as the primary sepsis study population.

## 3 Results

### 3.1 UPLC-MS analysis

**Figure 1:**
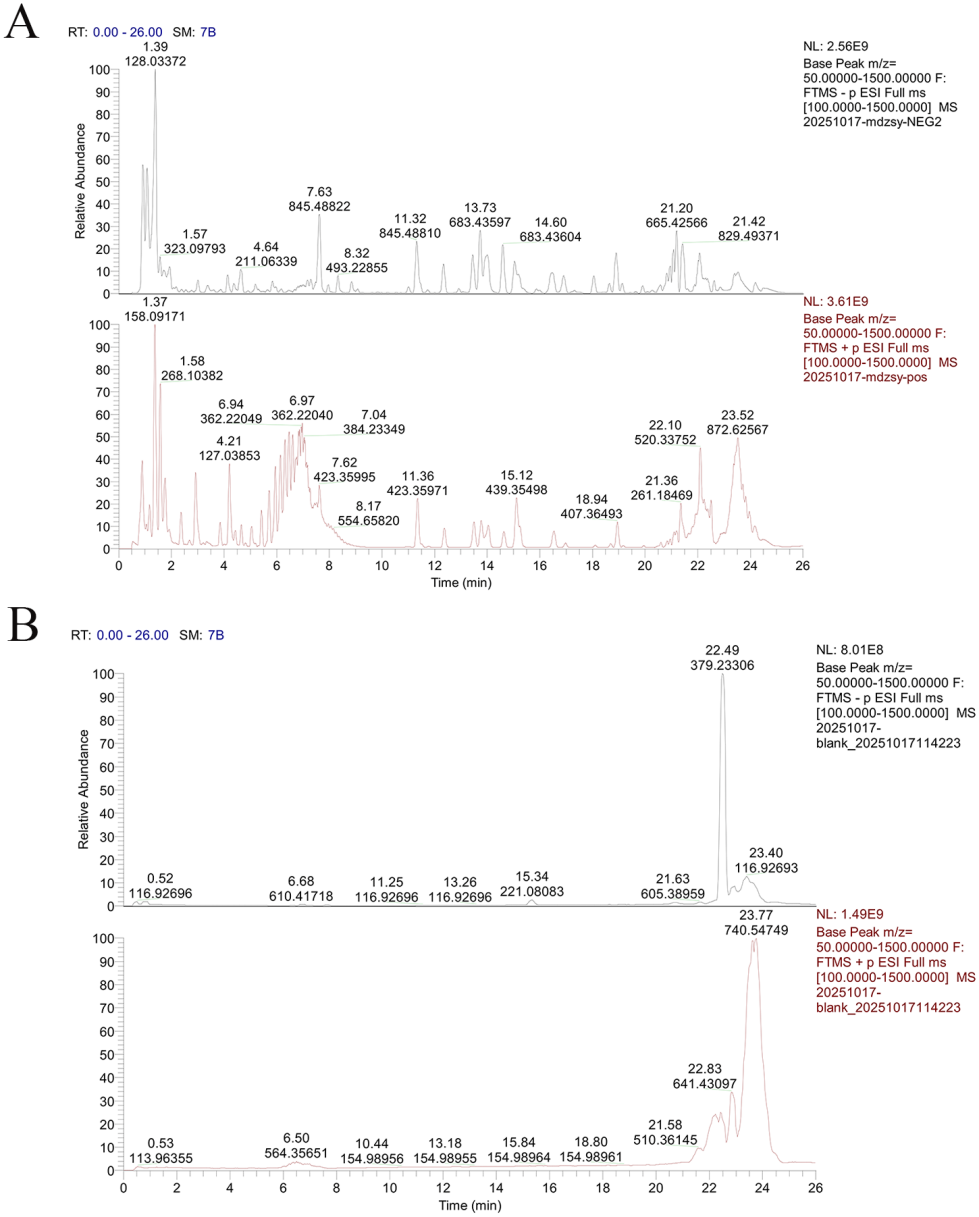
The total ion chromatograms of SMI and the reference blank preparation. **(A)** Total ion chromatograms of SMI; **(B)** Total ion chromatograms of reference blank preparation.

The chemical profiling of SMI was systematically characterized utilizing UPLC-MS. In total, 130 chemical components were identified and annotated, encompassing Ginsenoside Rb_1_, Ginsenoside Rg_1_, Ginsenoside Rg_3_, Schisandrin A, etc. (**Supplementary Table 1**). The representative total ion chromatograms (TICs) obtained from SMI and reference blank preparation are depicted in **Figure 1**.

### 3.2 Retrospective cohort study

**Figure 2:**
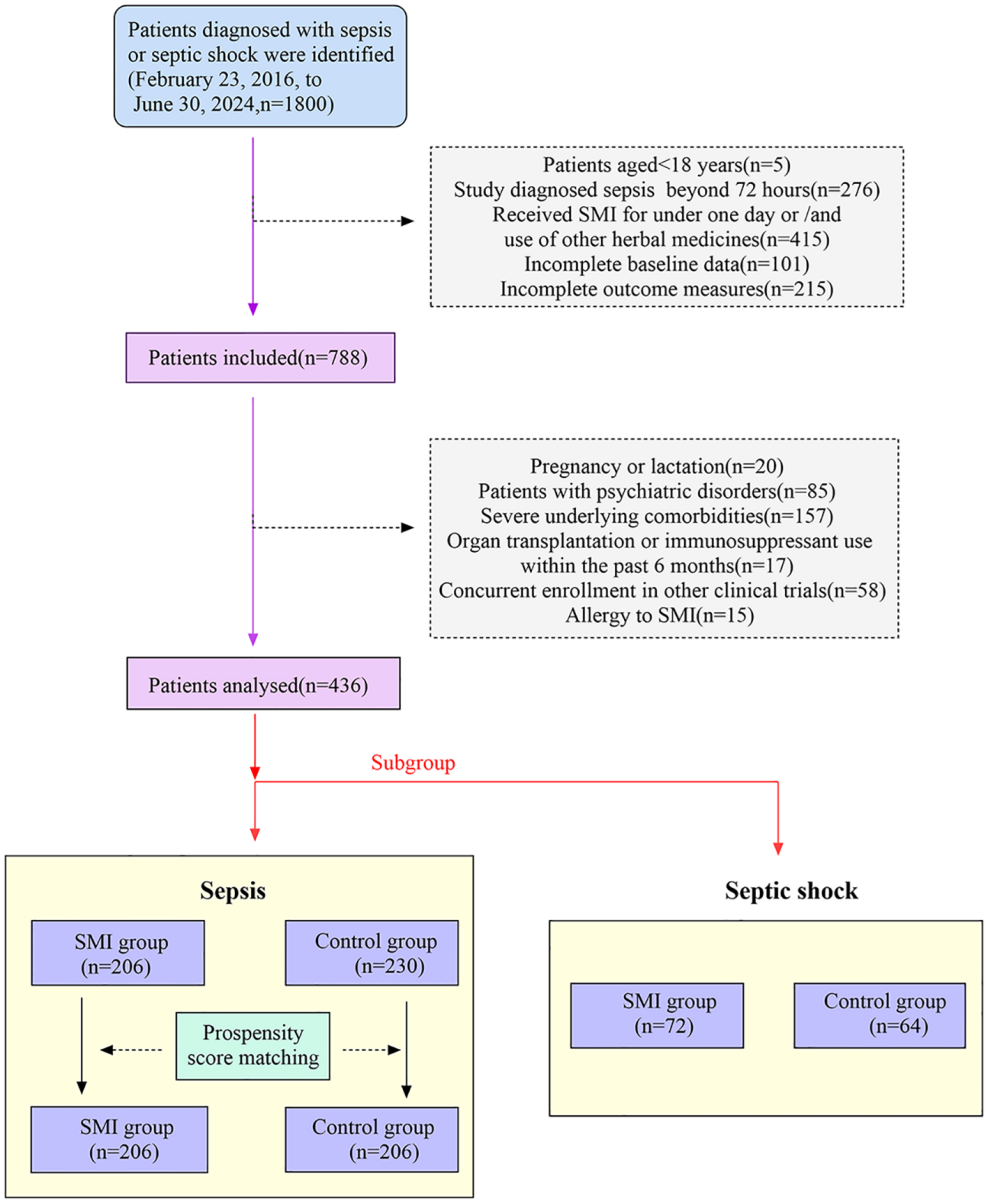
Flowchart of patient recruitment.

**Figure 3:**
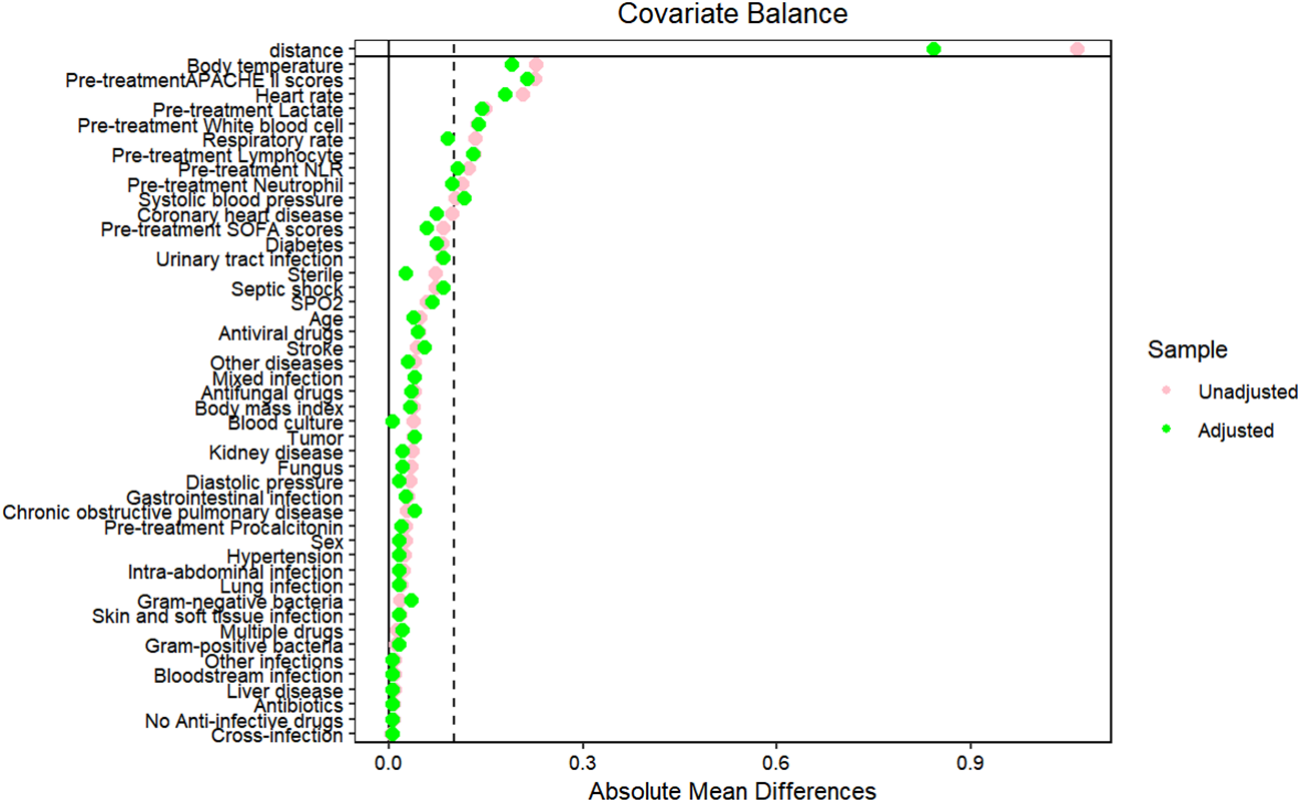
Baseline covariate balance in patients with sepsis. After 1:1 PSM, balanced cohorts of 206 patients per group were achieved for both study populations. Data points closer to the zero line indicate superior balance in baseline covariates.

According to pre-established inclusion and exclusion criteria, a total of 436 patients were enrolled after comprehensive screening. Among the enrolled population, 206 participants were assigned to the SMI group, while the remaining 230 were assigned to control group. The detailed patient enrollment workflow is illustrated in **Figure 2**. Initial comparative analyses demonstrated significant heterogeneity across baseline variables between the two groups, with notable discrepancies observed in heart rate and oxygen saturation (SpO_2_),and the prevalence of coronary heart disease. (**Table 1**). Accordingly, 1:1 PSM was conducted to eliminate confounding effects, yielding 206 matched individuals in each group. Post-matching results indicated improvements in intergroup covariate equilibrium (**Figure 3**). No significant intergroup distinction in 28-day mortality was identified between the two cohorts (*p* = 0.160). Under the assumption of no unmeasured confounding (*Γ* = 1.0), McNemar’s test yielded no significant between-group difference in 28-day mortality *(p* = 0.155).Rosenbaum bounds sensitivity analysis further demonstrated that the *Γ* value at which the upper-bound *p*-value exceeded 0.05 was roughly 1.1 (upper-bound *p* = 0.0945). This finding suggested that an unmeasured confounder that increased the odds of SMI administration by only 10% could offset the weak trend toward a survival benefit, thus reinforcing the reliability of our findings (**Supplementary Table 3**). In terms of secondary outcomes, the SMI group exhibited shorter ICU stays, reduced mechanical ventilation duration, and shorter febrile duration, along with lowered post-treatment clinical severity scores and biochemical markers, including SOFA scores, APACHE II scores, PCT, platelets, and blood lactate. Since most follow-up laboratory indicators remained within normal ranges, intergroup variations in lymphocyte, neutrophil, and NLR parameters should be interpreted with caution (**Table 2**).

**Table 1:** Baseline characteristics of the SMI and control groups before and after PSM in the Sepsis.

Characteristics	Before propensity score matching				After propensity score matching			
	SMI group (n= 206)	Control group (n=230)	*p* valve	SMD	SMI group (n= 206)	Control group (n=206)	*p* valve	SMD
Sex(Men)	114(55.30)	133(57.80)	0.601	0.050	114(55.30)	117(56.80)	0.766	0.029
Age	76.00(61.75-86.00)	76.00(68.00-85.00)	0.947	0.050	76.00(61.75-86.00)	76.00(69.00-85.00)	0.989	0.040
Body mass index	20.48(18.28-22.76)	20.60(18.30-22.93)	0.704	0.036	20.48(18.28-22.76)	20.60(18.30-22.90)	0.758	0.030
Vital signs								
Body temperature	36.80(36.50-37.50)	36.90(36.50-37.80)	0.132	0.203	36.80(36.50-37.50)	36.80(36.50-37.73)	0.328	0.169
Respiratory rate	20.00(20.00-23.00)	20.00(20.00-23.00)	0.780	0.146	20.00(20.00-23.00)	20.00(20.00-23.00)	0.908	0.098
Heart rate	89.00(80.00-103.25)	96.00(80.00-107.25)	0.036*	0.190	89.00(80.00-103.25)	95.00(80.00-107.25)	0.088	0.163
Systolic blood pressure	125.50(109.00-141.00)	124.50 (111.00-147.00)	0.295	0.101	125.50 (109.00-141.00)	126.00 (111.00-147.25)	0.250	0.114
Diastolic pressure	72.00(62.00-83.25)	70.50(62.00-82.25)	0.530	0.032	72.00(62.00-83.25)	71.00(62.00-83.00)	0.884	0.014
SPO_2_	98.00(96.00-99.00)	99.00(96.00-100.00)	0.000*	0.036	98.00(96.00-99.00)	99.00(96.00-100.00)	0.000*	0.042
Pre-treatment SOFA scores	4.00(3.00-8.00)	4.00(2.00-8.00)	0.407	0.086	4.00(3.00-8.00)	4.00(2.00-8.00)	0.521	0.058
Pre-treatment APACHE II scores	23.00(20.00-26.00)	22.00(19.00-25.00)	0.009*	0.210	23.00(20.00-26.00)	22.00(19.00-25.00)	0.015*	0.198
Present as septic shock	72(35.00)	64(27.80)	0.109	0.154	72(35.00)	55(26.70)	0.070	0.179
Source of infection								
Lung infection	156(75.70)	170(73.90)	0.663	0.042	156(75.70)	153(74.30)	0.733	0.034
Urinary tract infection	46(22.30)	70(30.40)	0.056	0.185	46(22.30)	63(30.60)	0.058	0.188
Skin and soft tissue infection	2(1.00)	6(2.60)	0.291	0.124	2(1.00)	5(2.40)	0.449	0.113
Intra-abdominal infection	8(3.90)	14(6.10)	0.294	0.101	8(3.90)	11(5.30)	0.481	0.069
Bloodstream infection	8(3.90)	7(3.00)	0.631	0.046	8(3.90)	7(3.40)	0.793	0.026
Gastrointestinal infection	23(11.20)	32(13.90)	0.388	0.083	23(11.20)	28(13.60)	0.454	0.074
Mixed infection	10(4.90)	20(8.70)	0.114	0.153	10(4.90)	18(8.70)	0.117	0.155
Other infections	8(3.90)	7(3.00)	0.631	0.046	8(3.90)	7(3.40)	0.793	0.026
Microorganisms								
Blood culture	90(43.70)	92(40.00)	0.435	0.075	90(43.70)	91(44.20)	0.921	0.010
Gram-negative bacteria	52(25.20)	62(27.00)	0.684	0.039	52(25.20)	59(28.60)	0.437	0.077
Gram-positive bacteria	25(12.10)	30(13.00)	0.776	0.027	25(12.10)	28(13.60)	0.659	0.044
Fungus	27(13.10)	38(16.50)	0.318	0.096	27(13.10)	23(11.20)	0.546	0.059
Cross-infection	13(6.30)	14(6.10)	0.923	0.009	13(6.30)	14(6.80)	0.842	0.020
Sterile	116(56.30)	113(49.10)	0.134	0.144	116(56.30)	111(53.90)	0.620	0.049
Anti-infective drugs								
Antibiotics	204(99.00)	229(99.60)	0.605	0.064	204(99.00)	205(99.50)	1.000	0.057
Antifungal drugs	58(28.20)	56(24.30)	0.366	0.087	58(28.20)	51(24.80)	0.434	0.077
Antiviral drugs	15(7.30)	27(11.70)	0.115	0.152	15(7.30)	24(11.70)	0.130	0.150
Multiple drugs	63(30.60)	73(31.70)	0.795	0.025	63(30.60)	67(32.50)	0.672	0.042
No Anti-infective drugs	2(1.00)	1(0.40)	0.605	0.064	2(1.00)	1(0.50)	1.000	0.057
Underlying comorbidities								
Hypertension	125(60.70)	145(63.00)	0.612	0.049	125(60.70)	128(62.10)	0.761	0.030
Coronary heart disease	40(19.40)	67(29.10)	0.019*	0.228	40(19.40)	55(26.70)	0.079	0.174
Diabetes	66(32.00)	55(23.90)	0.059	0.182	66(32.00)	51(24.80)	0.101	0.162
Stroke	45(21.80)	60(26.10)	0.301	0.100	45(21.80)	56(27.20)	0.208	0.124
Chronic obstructive pulmonary disease	41(19.90)	52(22.60)	0.491	0.066	41(19.90)	49(23.80)	0.340	0.094
Liver disease	49(23.80)	53(23.00)	0.855	0.018	49(23.80)	50(24.30)	0.908	0.011
Kidney disease	76(36.90)	93(40.40)	0.449	0.073	76(36.90)	80(38.80)	0.685	0.040
Tumor	20(9.70)	14(6.10)	0.159	0.135	20(9.70)	12(5.80)	0.141	0.145
Other diseases	147(71.40)	173(75.20)	0.363	0.087	147(71.40)	153(74.30)	0.506	0.066
Laboratory results								
Pre-treatment Procalcitonin	1.49(0.44-9.87)	1.55(0.35-8.35)	0.621	0.026	1.49(0.44-9.87)	1.48(0.34-8.35)	0.512	0.019
Pre-treatment White blood cell	13.85 (9.42-19.54)	13.00(10.12-17.47)	0.426	0.169	13.85(9.42-19.54)	12.90(10.22-17.37)	0.399	0.170
Pre-treatment Lymphocyte	1.03(0.76-1.77)	1.23(0.88-1.54)	0.194	0.170	1.03(0.76-1.77)	1.23(0.88-1.54)	0.232	0.167
Pre-treatment Neutrophil	11.91(7.12-17.42)	10.76(8.97-14.25)	0.842	0.145	11.91(7.12-17.42)	10.87(9.05-14.43)	0.947	0.126
Pre-treatment Platelet	190.50(104.75-303.25)	196.50 (165.00-254.00)	0.452	0.060	190.50 (104.75-303.25)	197.00(164.00-254.00)	0.596	0.083
Pre-treatment NLR	9.67(5.26-16.35)	9.12(5.82-13.91)	0.918	0.157	9.67(5.26-16.35)	9.21(5.82-14.79)	0.908	0.134
Pre-treatment Lactate	2.37(1.65-3.30)	1.90(1.30-2.90)	0.001*	0.133	2.37(1.65-3.30)	1.90(1.30-2.90)	0.002*	0.126
Medication course	50.00(40.00-50.00)	0.00	0.000*		4.00(2.00-6.00)	0.00	0.000*	
Drug dosage	4.00(2.00-6.00)	0.00	0.000*		50.00(40.00-50.00)	0.00	0.000*	

*p<0.05.Categorical data were expressed as frequency and percentage [n (%)], normally distributed data were presented as mean ± standard deviation(SD), and non-normal data were expressed as median and interquartile range (IQR).SMI, Shengmai Injection; PSM, Propensity score matching; SPO_2_, Peripheral Capillary Oxygen Saturation; SOFA, Sequential Organ Failure Assessment; APACHE, Acute Physiology and Chronic Health Evaluation; NLR, Neutrophil-to-lymphocyte Ratio.

**Table 2:** Comparative outcomes between the SMI and control groups in sepsis.

Outcome	SMI group(n=206)	Control group (n=206)	*P* value
28-day mortality	32(15.50)	43(20.90)	0.160
Length of ICU stay	0.00(0.00-10.00)	4.00(0.00-13.25)	0.017*
Length of hospital stay	16.00 (10.00-27.25)	16.00 (11.00-27.25)	0.734
Duration of ventilator use	0.00 (0.00-294.89)	21.98 (0.00-334.75)	0.035*
Duration of invasive ventilator	0.00(0.00-0.00)	0.00(0.00-42.45)	0.000*
Duration of non-invasive ventilator	0.00 (0.00-181.83)	0.00 (0.00-63.00)	0.473
Duration of CRRT	0.00 (0.00-0.00)	0.00 (0.00-0.00)	0.000*
Post-treatment SOFA scores	4.00 (2.00-5.00)	4.00 (3.00-6.00)	0.003*
Post-treatment APACHE II scores	12.00 (10.00-14.00)	13.00 (10.75-16.00)	0.003*
Post-treatment Procalcitonin	0.67 (0.18-2.68)	1.09 (0.35-4.67)	0.014*
Post-treatment White blood cell	8.65(5.78-10.76)	9.47(6.20-10.87)	0.055
Post-treatment Platelet	165.00 (107.75-267.00)	194.50 (164.00-235.75)	0.013*
Post-treatment Lymphocyte	1.23(0.97-1.76)	1.43(0.99-1.76)	0.016*
Post-treatment Neutrophil	5.44(3.63-7.88)	4.54(3.63-5.64)	0.003*
Post-treatment NLR	4.12(2.46-6.68)	3.28(2.42-4.77)	0.004*
Post-treatment Lactate	1.69(1.22-2.40)	2.03(1.40-2.56)	0.004*
Fever duration	3.00(2.00-6.00)	4.00(1.00-10.00)	0.039*

*p<0.05. Categorical data were expressed as frequency and percentage [n (%)], normally distributed data were presented as mean ± standard deviation(SD), and non-normal data were expressed as median and interquartile range (IQR).SMI, Shengmai Injection; ICU, Intensive care unit; CRRT, Continuous renal replacement therapy; SOFA, Sequential Organ Failure Assessment; APACHE, Acute Physiology and Chronic Health Evaluation; NLR, Neutrophil-to-lymphocyte Ratio.

**Figure 4:**
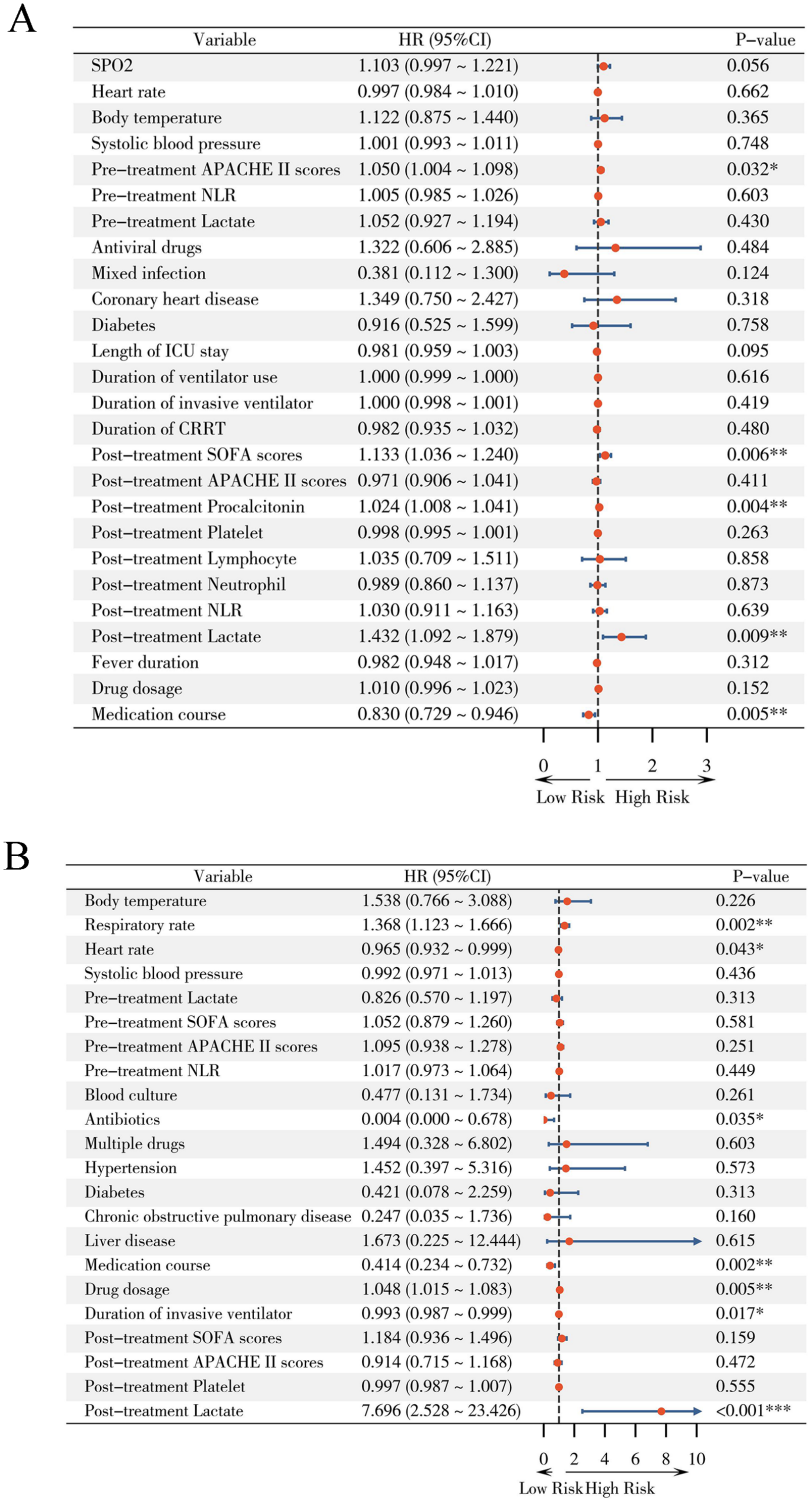
Cox regression analysis for 28-day mortality in sepsis and septic shock. **(A)** Cox regression analysis for 28-day mortality in sepsis; **(B)** Cox regression analysis for 28-day mortality in septic shock. *p < 0.05, **p < 0.01; ***p < 0.001.

**Figure 5:**
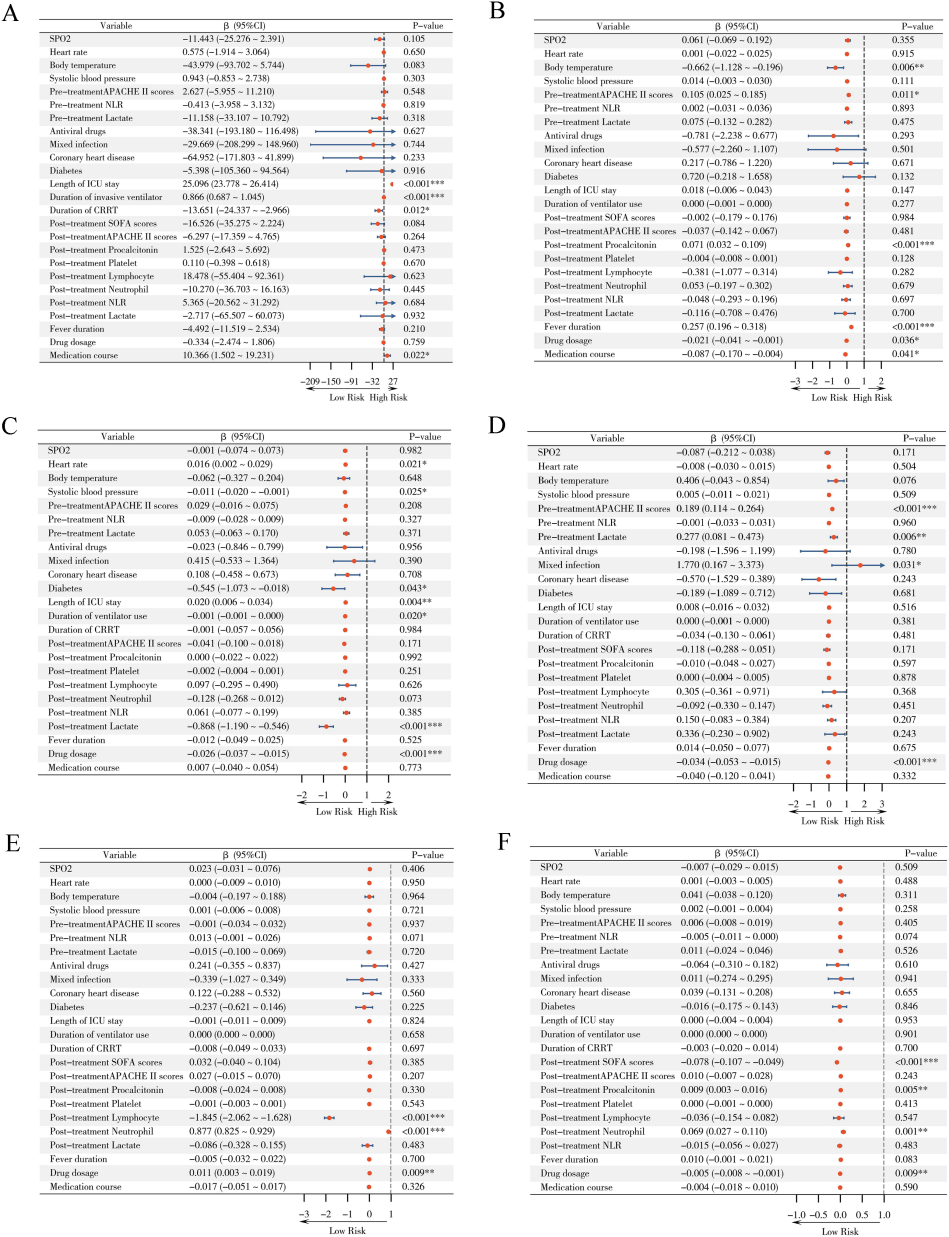
Linear regression analyses of variables associated with SMI dosage or medication course with significant differences in sepsis. Statistically significant secondary outcomes from the univariate analysis were used as dependent variables. **(A)** Linear regression results for Duration of ventilator use; **(B)** Linear regression results for Duration of CRRT; **(C)** Linear regression results for Post-treatment SOFA scores; **(D)** Linear regression results for Post-treatment APACHE II scores; **(E)** Linear regression results for Post-treatment NLR; **(F)** Linear regression results for Post-treatment Lactate. *p < 0.05; **p < 0.01; ***p < 0.001.

Multivariable Cox regression analysis targeting 28-day mortality as the primary endpoint revealed that post-treatment SOFA scores (HR = 1.133, 95% CI = 1.036 to 1.240), PCT (HR = 1.024, 95% CI = 1.008 to 1.041), lactate levels (HR = 1.432, 95% CI = 1.092 to 1.879), and SMI treatment course (HR = 0.830, 95%CI=0.729 to 0.946) were statistically significant factors (**Figure 4A**). Subsequent linear regression analyses demonstrated that duration of CRRT (β=-0.021,95%CI=-0.041 to -0.001),post-treatment SOFA scores (β=-0.026, 95%CI=-0.037 to -0.015),APACHE II scores(β=-0.034, 95%CI=-0.053 to -0.015), NLR(β=0.011, 95%CI= 0.003 to 0.019), and lactate levels(β=-0.005, 95%CI=-0.008 to -0.001) were independently associated with SMI dosage. Meanwhile, ventilator duration(β=10.366, 95%CI=1.502 to 19.231), and CRRT duration (β=-0.087, 95%CI=-0.170 to -0.004) were correlated with the course of SMI intervention (**Figures 5A–F**; **Supplementary Figures S1–1**-**S1-7**). Collectively, SMI intervention reduced ICU length of stay, ventilation duration, and febrile duration, along with notable reductions in post-treatment SOFA scores, APACHE II scores, PCT concentrations, platelet counts, and blood lactate levels. Furthermore, SOFA scores, PCT, and circulating lactate were identified as independent predictors for 28-day mortality among septic patients.

**Figure 6:**
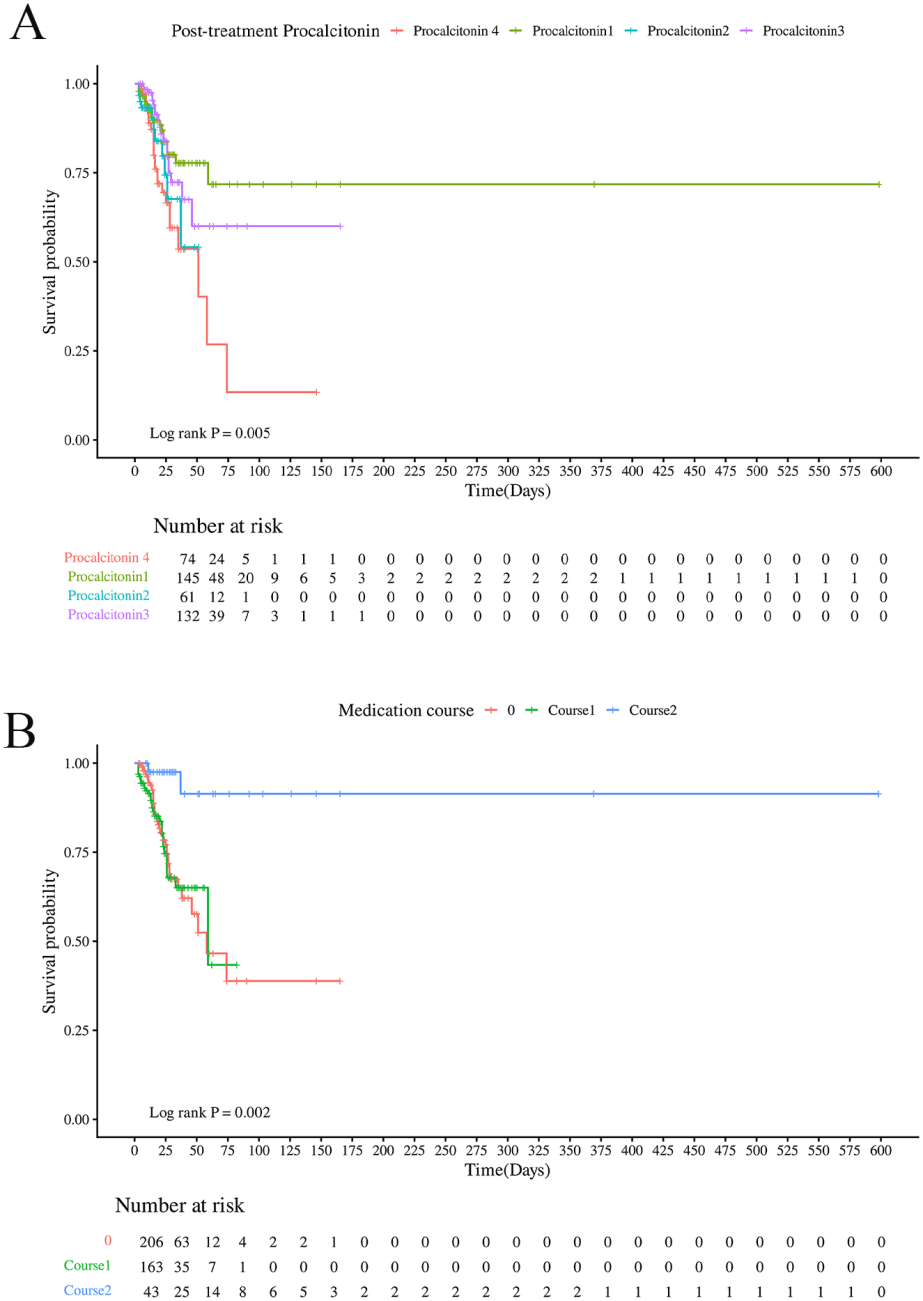
Kaplan–Meier survival curves of variables with significant differences in sepsis. **(A)** Kaplan-Meier survival curves for Post-treatment PCT (*p* = 0.005); **(B)** Kaplan-Meier survival curves for SMI medication course(*p* = 0.002). Procalcitonin 1:SMI group + PCT < 2 ng/mL; Procalcitonin 2:SMI group + PCT ≥2 ng/mL; Procalcitonin 3: Control group + PCT < 2 ng/mL; Procalcitonin 4: Control group + PCT ≥2 ng/mL. Course 1: 0<Medication course<7 days; Course 2: Medication course ≥7 days.

Stratified subgroup survival analyses were implemented to evaluate the prognostic significance of pivotal variables in sepsis, with age and treatment course included based on clinical plausibility. According to Kaplan-Meier survival analysis, no marked intergroup survival differences were observed across grouping, age strata, SMI dosage, post-treatment SOFA scores, and lactate concentrations. Conversely, post-treatment PCT (*p* = 0.005) and medication course(*p* = 0.002) remained independent prognostic indicators. Importantly, superior survival rates were observed in the SMI cohort among individuals with post-treatment PCT below 2 ng/mL or prolonged SMI therapy of no less than 7 days (**Figure 6A**; **Supplementary Figures S2-1**–**S2-5**).

**Figure 7:**
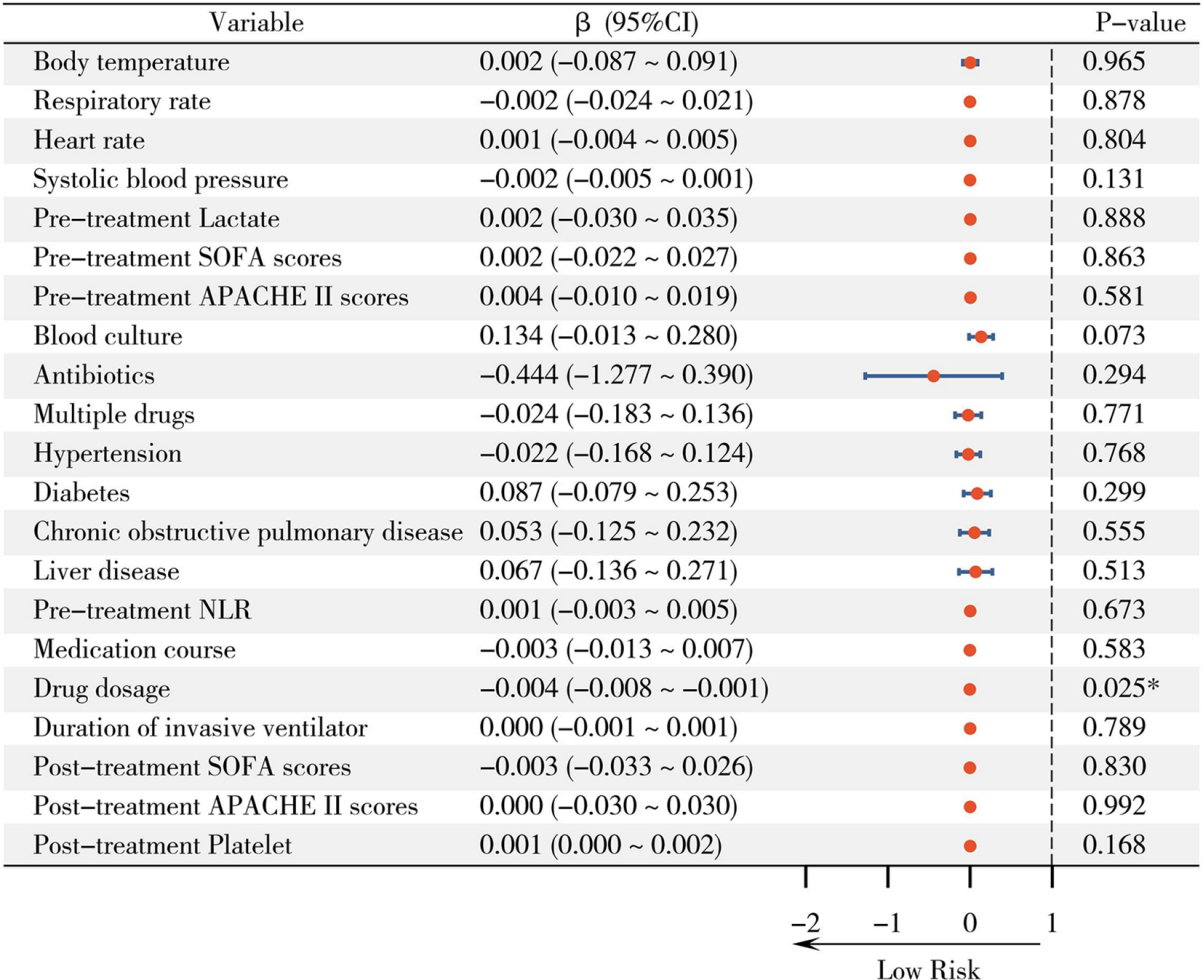
Linear regression analyses of post-treatment lactate associated with SMI dosage or medication course with significant differences in septic shock. Statistically significant secondary outcomes from the univariate analysis were used as dependent variables *p < 0.05.

A subgroup analysis of septic shock, a life-threatening severe phenotype within the sepsis disease spectrum, was performed to evaluate the efficacy of SMI. In the study,72 patients were enrolled in the SMI group and 64 in the control group. Nevertheless, the septic shock cohort still exhibited several imbalanced variables with an SMD greater than 0.2 even after multiple matching strategies were applied. Given methodological concerns regarding over-matching, PSM was not performed for the septic shock group(**Table 3**). Between-group comparison analysis indicated that SMI treatment significantly reduced the duration of invasive ventilation, decreased platelet and lactate levels, and mitigated post-treatment SOFA and APACHE II scores in septic shock (**Table 4**). Multivariable Cox regression revealed that post-treatment lactate, medication course, duration of invasive ventilator, and SMI dosage served as independent predictors for 28-day mortality (HR = 7.696, 95% CI = 2.528 to 23.426; HR = 0.414, 95% CI = 0.234 to 0.732;HR=0.993, 95% CI = 0.987 to 0.999;HR=1.048, 95% CI = 1.015 to 1.083). Moreover, linear regression analysis indicated significant associations between SMI dosage and post-treatment lactate levels(β=-0.004, 95% CI=-0.008 to -0.001) (**Figure 7**; **Supplementary Figures S3-1**–**S3-4**). In summary, SMI therapy conferred clinical benefits by reducing the duration of invasive ventilation, improving severity scores, and decreasing platelet and lactate levels. In contrast, no variables, including age, treatment group, SMI dosage, intervention duration, post-treatment SOFA, lactate, and PCT, were identified as significant prognostic factors via Kaplan-Meier analysis among patients with septic shock (**Supplementary Figures S4-1**–**S4-7**).

**Table 3:** Baseline characteristics of the SMI and control groups in the Septic shock.

Characteristics	SMI group (n= 72)	Control group (n=64)	P valve
Sex(Men)	37(51.40)	36(56.30)	0.570
Age	77.00(61.00-86.00)	75.00(69.00-83.00)	0.639
Body mass index	20.73(18.68-22.97)	20.80(18.40-23.00)	0.970
Vital signs			
Body temperature	36.80(36.50-37.50)	36.90(36.50-37.83)	0.904
Respiratory rate	20.00(20.00-23.00)	20.00(20.00-23.00)	0.585
Heart rate	88.00(80.25-102.75)	98.00(84.25-112.50)	0.053
Systolic blood pressure	122.50(95.25-141.75)	121.50 (102.50-144.00)	0.270
Diastolic pressure	69.00(58.00-78.00)	68.00(57.25-77.75)	0.937
SPO2	98.00(97.00-99.00)	98.00(96.25-100.00)	0.234
Pre-treatment SOFA scores	6.00(3.00-10.00)	4.00(2.00-7.00)	0.058
Pre-treatment APACHE II scores	23.00(20.00-26.00)	22.00(20.00-24.75)	0.132
Source of infection			
Lung infection	55(76.40)	51(79.70)	0.643
Urinary tract infection	12(16.70)	18(28.10)	0.108
Skin and soft tissue infection	0.00	2(3.10)	0.220
Intra-abdominal infection	3(4.20)	2(3.10)	1.000
Bloodstream infection	0.00	1(1.60)	0.471
Gastrointestinal infection	8(11.10)	13(20.30)	0.138
Mixed infection	3(4.20)	4(6.30)	0.706
Other infections	5(6.90)	3(4.70)	0.722
Microorganisms			
Blood culture	34(47.20)	24(37.50)	0.253
Gram-negative bacteria	16(22.20)	15(23.40)	0.866
Gram-positive bacteria	9(12.50)	10(15.60)	0.600
Fungus	13(18.10)	14(21.90)	0.577
Cross-infection	4(5.60)	5(7.80)	0.734
Sterile	38(52.80)	30(46.90)	0.492
Anti-infective drugs			
Antibiotics	71(98.60)	64(100.00)	1.000
Antifungal drugs	20(27.80)	19(29.70)	0.806
Antiviral drugs	3(4.20)	7(10.90)	0.238
Multiple drugs	21(29.20)	22(34.40)	0.514
No Anti-infective drugs	1(1.40)	0.00	1.000
Underlying comorbidities			
Hypertension	37(51.40)	39(60.90)	0.263
Coronary heart disease	19(26.40)	17(26.60)	0.982
Diabetes	24(33.30)	16(25.00)	0.287
Stroke	13(18.10)	14(21.90)	0.577
Chronic obstructive pulmonary disease	11(15.30)	15(23.40)	0.227
Liver disease	11(15.30)	15(23.40)	0.227
Kidney disease	21(29.20)	21(32.80)	0.646
Tumor	3(4.20)	4(6.30)	0.706
Other diseases	49(68.10)	52(81.30)	0.079
Laboratory results			
Pre-treatment Procalcitonin	1.76(0.49-11.58)	1.28(0.27-5.49)	0.552
Pre-treatment White blood cell	14.20(9.09-20.07)	12.18(9.39-19.10)	0.427
Pre-treatment Lymphocyte	1.02(0.79-1.75)	1.30(0.89-1.54)	0.480
Pre-treatment Neutrophil	13.02(7.97-17.89)	10.60(8.79-13.30)	0.249
Pre-treatment Platelet	189.50 (104.50-287.25)	198.00(165.50-261.50)	0.624
Pre-treatment NLR	9.20(5.11-16.42)	8.90(5.82-12.09)	0.744
Pre-treatment Lactate	2.35(1.50-3.45)	1.75(1.30-2.60)	0.037*
Medication course	3.50(2.00-6.00)	0.00	0.000*
Drug dosage	50.00(40.00-50.00)	0.00	0.000*

*p<0.05.Categorical data were expressed as frequency and percentage [n (%)], normally distributed data were presented as mean ± standard deviation(SD), and non-normal data were expressed as median and interquartile range (IQR).SMI, Shengmai Injection; SPO_2_, Peripheral Capillary Oxygen Saturation; SOFA, Sequential Organ Failure Assessment; APACHE, Acute Physiology and Chronic Health Evaluation; NLR, Neutrophil-to-lymphocyte Ratio.

**Table 4:** Comparative outcomes between the SMI and control groups in septic shock.

Outcome	SMI group (n=72)	Control group (n=64)	*P* value
28-day mortality	10(13.89)	9(14.10)	0.977
Length of ICU stay	0.00(0.00-7.00)	2.00(0.00-13.00)	0.406
Length of hospital stay	15.00(10.00-27.50)	15.50(9.00-21.75)	0.591
Duration of ventilator use	0.00(0.00-135.63)	12.13(0.00-332.21)	0.214
Duration of invasive ventilator	0.00(0.00-0.00)	0.00(0.00-7.27)	0.000*
Duration of non-invasive ventilator	0.00(0.00-124.17)	0.00(0.00-68.63)	0.798
Duration of CRRT	0.00(0.00-0.00)	0.00(0.00-0.00)	0.060
Post-treatment SOFA scores	5.00(4.00-6.00)	6.00(4.00-7.75)	0.000*
Post-treatment APACHE II scores	12.00(10.00-13.00)	12.00(11.00-15.00)	0.017*
Post-treatment Procalcitonin	0.59(0.18-2.21)	1.05(0.39-3.58)	0.153
Post-treatment White blood cell	5.76(4.79-6.62)	5.57(4.77-7.21)	0.724
Post-treatment Platelet	165.00(106.25-244.00)	195.00(164.25-237.25)	0.024*
Post-treatment Lymphocyte	1.21(0.96-1.63)	1.38(1.00-1.76)	0.154
Post-treatment Neutrophil	4.76(3.58-5.71)	4.45(3.65-5.43)	0.571
Post-treatment NLR	3.76(2.20-4.86)	3.36(2.35-4.35)	0.524
Post-treatment Lactate	1.24(1.08-1.42)	1.32(1.20-1.60)	0.001*
Fever duration	3.00(1.00-6.75)	3.00(0.00-8.00)	0.597

*p<0.05.Categorical data were expressed as frequency and percentage [n (%)], normally distributed data were presented as mean ± standard deviation (SD), and non-normal data were expressed as median and interquartile range (IQR).SMI, Shengmai Injection; ICU, Intensive care unit; CRRT, Continuous renal replacement therapy; SOFA, Sequential Organ Failure Assessment; APACHE, Acute Physiology and Chronic Health Evaluation; NLR, Neutrophil-to-lymphocyte Ratio.

## 4 Discussion

This multicenter retrospective study enrolled 426 sepsis patients (n = 136 with septic shock) to comprehensively assess the clinical efficacy and prognostic value of SMI therapy. Following 1:1 PSM, a balanced cohort was established (n = 206 per group), further substantiating the protective effects of SMI intervention. Specifically, SMI treatment was associated with decreased ICU length of stay, reduced mechanical ventilation duration, and diminished CRRT duration. In addition, SMI therapy improved post-treatment inflammatory and metabolic profiles, accompanied by declines in SOFA and APACHE II scores, as well as in PCT and lactate levels. Although several earlier small-sample and low-quality clinical studies reported significant reductions in 28-day mortality among patients with sepsis (Huang et al., 2011; Yang et al., 2021; Yao et al., 2021), the majority of existing investigations yielded neutral non-significant outcomes (Chen et al., 2015a; Guo et al., 2010). Furthermore, the latest comprehensive meta-analysis incorporating 17 RCTs involving 860 enrolled participants (Ha et al., 2019), together with accumulating published evidence, indicated that SMI does not reduce 28-day mortality in septic patients, which is highly consistent with the findings of our present study. Compared with previously published relevant investigations, the present work delivers incremental scientific advances across methodological design, statistical strategy, clinical observation, and mechanistic exploration, which are elaborated as follows. First, this study represents the largest retrospective cohort investigation to date focusing on the therapeutic effects of SMI in patients with sepsis and septic shock. Second, a broad panel of clinical endpoints was incorporated to comprehensively validate the clinical efficacy of SMI. Third, this study systematically dissected the multifaceted clinical benefits of SMI during sepsis progression, including protective effects on multiple organ function, alleviation of excessive inflammatory response, and improvement of metabolic derangements. Fourth, separate multivariate prognostic models were constructed to quantitatively analyze the predictive relevance of SMI intervention regarding secondary outcome endpoints. Fifth, a rigorous multicenter real-world cohort study was conducted with PSM for adjustment for confounders, followed by sensitivity analyses to assess the robustness of our findings. Finally, profiling the chemical composition of SMI established a potential causal link between its active ingredient spectrum and its observed therapeutic effects, establishing a material foundation for its mechanism of action against sepsis. While SMI intervention did not significantly improve the primary endpoint of 28-day mortality, it significantly ameliorated secondary outcomes involving inflammatory, metabolic, and organ function aberrations. These findings offer valuable clinical implications for sepsis and septic shock management; however, large-scale prospective RCTs and further mechanistic studies are required to validate these observational conclusions.

Notably, multivariable regression identified post-treatment PCT and lactate levels as independent predictors of 28-day mortality in sepsis. Favorable survival outcomes were evident in SMI-treated patients with PCT < 2 ng/mL. Correspondingly, among patients with septic shock, SMI therapy was associated with mitigated elevated post-treatment SOFA and APACHE II scores alongside reduced lactate levels. These findings collectively suggest that SMI represents a promising adjunctive therapeutic candidate for severe sepsis and septic shock by modulating inflammatory pathophysiological cascades and thereby improving clinical prognosis. Collectively, these clinical observations support the potential of SMI to preserve organ function, suppress inflammatory responses, and ameliorate metabolic dysregulation in patients with sepsis and septic shock. Such clinical benefits may be attributed to the multifaceted pharmacological profiles of SMI, which act on core pathological pathways that drive the progression of septic pathophysiology.

Ginsenosides, the primary bioactive constituents derived from *Panax ginseng* C. A. Meyer (Hong Shen), with ginsenoside Rb_1_, Rg_1_, and Rg_3_ as representative monomers, exert notable anti-inflammatory, immunoregulatory, and antioxidant effects. Ginsenoside Rb_1_ suppresses NF-κB cascade activation to limit pro-inflammatory cytokine release, constrains ferroptosis to ameliorate septic pathogenesis, and maintains pulmonary AT2 cell homeostasis to alleviate sepsis-associated acute lung injury (Gao et al., 2026; He et al., 2025; Yu et al., 2023). Ginsenoside Rg_1_ modulates the Prdx1-PTEN/PI3K/AKT signaling pathway to alleviate sepsis-associated acute respiratory distress syndrome and downregulates the expression of TLR4, NF-κB, and NLRP3 inflammatory complexes to restore cardiac contractile function (Luo et al., 2020; Xue et al., 2026). Ginsenoside Rg_3_ suppresses inflammatory cascades by inhibiting NF-κB activation and subsequent pro-inflammatory cytokine production, potentiates antioxidant defenses through activation of Nrf2, and enhances immune homeostasis by modulating immune cell profiles and blunting PD-L1 glycosylation (Yao and Zhu, 2025).

It has been reported that *Schisandra chinensis* Turcz. (Baill.) (Wu Weizi) possesses abundant phenolic acids, triterpenoids, and lignans, which collectively exert prominent anti-inflammatory activities by inhibiting iNOS and COX-2 expression and suppressing AKT/NF-κB signaling (Kopustinskiene and Bernatoniene, 2021). Ophiopogonis japonicus polysaccharides (OJPs), the core bioactive macromolecules derived from *Ophiopogon japonicus* (Mai Dong), exhibit immunoregulatory, antioxidant, cardioprotective, and antitumor properties. They mitigate inflammatory injury through blockade of the TLR4/MyD88/NF-κB, ameliorate intracellular oxidative stress by orchestrating the Nrf2/Keap1 and FNIP1/FEM1B axes, and strengthen immune cell function and immunoglobulin secretion to reshape host immune homeostasis (Ding et al., 2025; Wang et al., 2023). Although the therapeutic efficacy of SMI in sepsis is supported by multiple anti-inflammatory and antioxidant mechanisms, the current evidence is largely preclinical, and these mechanisms remain hypothetical until confirmed through rigorous prospective validation.

Corroborating these pharmacological mechanisms, PCT and lactate, as core inflammatory and metabolic markers, hold substantial clinical relevance for reflecting disease severity and predicting survival among septic patients.

PCT, a glycoprotein derived from thyroid C cells, represents an ideal biomarker with superior sensitivity and specificity for bacterial infection identification, and is extensively utilized for clinical diagnostic evaluation and septic severity stratification (Shang et al., 2022). Circulating PCT upregulation correlates positively with the severity of acute infection. Furthermore, substantial evidence elucidated that heightened PCT levels are closely intertwined with dysregulated immune function and pathological inflammatory cascades in sepsis, thereby validating PCT as a robust biomarker for septic severity evaluation and prognostic stratification (Lai et al., 2024). In addition to its diagnostic and prognostic significance, PCT-directed antimicrobial management yields remarkable clinical benefits for critically ill individuals. A meta-analysis has confirmed that optimized antibiotic regimens guided by PCT enhanced survival and reduced antibiotic exposure duration in ICU patients with infection (Wirz et al., 2018). Based on the foregoing findings, pooled clinical analyses have established that PCT algorithm-directed antibiotic withdrawal regimes achieve a 2.0-day reduction in cumulative antibiotic exposure duration (moderate-certainty evidence) versus conventional standard-of-care management, accompanied by a 5% decline in both short-term and long-term mortality risk (moderate-certainty evidence) (Rafiq et al., 2026).

In terms of predicting septic mortality, prospective data from a 421-patient cohort revealed that PCT surpasses human neutrophil lipocalin (HNL), CRP, and peripheral leukocyte count in discriminating bacterial sepsis (Benhamou et al., 2025) Besides, a large-scale cohort study (n=2492) emphasized that continuous PCT monitoring is indispensable for clinicians to identify patients at high risk of developing severe sepsis and administer timely, targeted interventions (Wang et al., 2024). Despite its well-established value in stratifying sepsis severity, distinguishing bacterial infections from viral etiologies, and optimizing the duration of antibacterial therapy, PCT exhibits inherent non-specificity and suboptimal absolute sensitivity, thereby necessitating prudent clinical interpretation. Accordingly, PCT should serve as an adjunctive, not definitive, biomarker in sepsis and septic shock, interpreted in conjunction with clinical evaluation and other lab findings.

Circulating lactate is a pivotal biomarker of tissue hypoxia and microcirculatory dysfunction, with substantial prognostic significance in sepsis and septic shock. Hyperlactatemia was defined as a blood lactate concentration ≥2 mmol/L, while lactic acidosis was diagnosed at concentrations≥4 mmol/L (Müller et al., 2023). Serum lactate concentrations are positively correlated with SOFA scores, and a lactate threshold exceeding 3 mmol/L serves as an independent prognostic predictor of 30-day mortality (OR = 2.200, *p* = 0.009), with advancing age further exacerbating the associated mortality risk (Andersson et al., 2025; Oh et al., 2019). Beyond its utility as a metabolic biomarker utility, lactate functions as a key immunomodulator in disease pathogenesis. It inhibits T-cell activation through the CD40LG/SOCS3-JAK1/STAT3 axis, facilitates GPR81-mediated and epigenetically regulated polarization of macrophages toward the anti-inflammatory M2 phenotype, and induces tubular epithelial cell pyroptosis via H3K18 lactylation-dependent SPHK1–SIRT1 signaling. These combined actions ultimately lead to immunosuppression and organ dysfunction (Certo et al., 2021; Chen et al., 2025; Huang et al., 2026; Zhang et al., 2025).

In addition to its clinical value in diagnosis and prognosis, lactate exerts independent bioactive effects, thereby emerging as a promising therapeutic candidate beyond its role as a metabolic stress byproduct. Experimental findings revealed that hypertonic sodium lactate, relative to saline, improves mesenteric microperfusion, cardiac contractile performance, and fractional shortening (Tchatat Wangueu et al., 2025). An *in vitro* study showed that lactate inhibits NF-κB signaling and mast cell degranulation, thereby dampening inflammatory responses during the immunosuppressive phase of sepsis (Yang et al., 2020). Lactate serves as both a core indicator of compromised tissue perfusion and a central modulator of host immunity in sepsis. However, due to its poor specificity for infectious etiology, reliable prognostic stratification requires the integrated assessment of lactate profiles alongside diverse biomarkers, organ function metrics, and clinical manifestations.

Although robust clinical and pharmacological evidence corroborates the therapeutic potential of SMI in sepsis and septic shock, the present study bears notable limitations. While independent prognostic predictors of 28-day mortality were identified, the absence of a significant survival benefit with SMI necessitates cautious interpretation of the clinical findings. The *post-hoc* power analysis and sample size calculation (*w* = 0.0992, critical χ²= 3.84146, power = 0.5214, n = 798; **Supplementary Figure 5**) indicated that the sample size was insufficient to detect a modest yet clinically significant effect. A 28-day follow-up may be insufficient to capture the full impact of SMI, which might manifest as improved long-term recovery and reduced post-sepsis morbidity rather than short-term survival. This multicenter retrospective observational design carries inherent residual selection bias and confounding, even following PSM. To some extent, complete-case analysis in the inclusion criteria can reduce statistical power, inflate standard errors, and discard partially observed data, thereby compromising efficiency when missingness is associated with exposure or outcome and differs systematically from the included studies. From a methodological perspective, covariate balance constitutes a large-sample statistical property. PSM inevitably reduces the effective sample size, thereby attenuating statistical power to identify residual intergroup imbalance; accordingly, PSM cannot guarantee optimal balance across all measured covariates. SMI dosage, timing, and treatment duration adhered to routine clinical practice instead of unified protocols, inducing interindividual heterogeneity. Moreover, the absence of serial biomarker kinetics and mechanistic analyses limits the ability to draw conclusions about causal relationships between SMI−mediated effects and clinical outcomes. Additionally, heterogeneity in sample sources, limited sample quantities, the absence of serial dynamic assessments of endpoints, and discrepancies in medication dosages and treatment courses further restrict the generalizability and robustness of our findings.

Nevertheless, this study offers valuable insights into the prognostic and therapeutic value of inflammatory and metabolic biomarkers in sepsis. Post-treatment PCT and lactate were validated as independent predictors of 28-day mortality, underscoring their essential utility in risk stratification and therapeutic surveillance. Crucially, the present analysis validated that adjunctive SMI therapy lowers PCT, lactate, and SOFA scores, verifying its capacity to mitigate excessive inflammation, restore metabolic homeostasis, and preserve organ function. These observations aligned with the multi−target pharmacological activities of ginsenosides, lignans, and polysaccharides in SMI, which collectively modulate inflammatory signaling, oxidative stress, and immune homeostasis. Future prospective, RCTs with standardized protocols and serial biomarker monitoring are warranted to validate the efficacy of SMI and elucidate its precise mechanisms of action.

## 5 Conclusion

In conclusion, SMI treatment was associated with favorable physiological and biochemical changes in patients with sepsis, as evidenced by shortened ICU stays, reduced ventilation and fever durations, and improved clinical markers, including SOFA and APACHE II scores, PCT, lactate, and platelet counts. Although 28-day mortality was not significantly reduced, the therapy was beneficial in septic shock, where it further shortened invasive ventilation, decreased lactate and platelet levels, and mitigated post-treatment SOFA and APACHE II scores. The absence of a clear survival advantage may be related to limited sample size, the retrospective observational design, or the possibility that SMI improves systemic physiological parameters without directly modulating the core pathogenic pathways that drive mortality in critical illness. These effects may be attributable to the multi-target activities of SMI’s bioactive constituents. Large-scale prospective RCTs and rigorous mechanistic studies are needed to validate the clinical efficacy of SMI and elucidate its underlying molecular mechanisms.

## Data Availability

The raw data supporting the conclusions of this article will be made available by the authors, without undue reservation.

## References

[B1] Andersson M. Fröderberg Schooner K. Karlsson Werther V. Karlsson T. De Geer L. Wilhelms D. B. . (2025). Prehospital lactate analysis in suspected sepsis improves detection of patients with increased mortality risk: an observational study. Crit. Care 29, 38. doi: 10.1186/s13054-024-05225-2 39838391 PMC11753079

[B2] Austin P. C. (2009). Balance diagnostics for comparing the distribution of baseline covariates between treatment groups in propensity-score matched samples. Stat. Med. 28, 3083–3107. doi: 10.1002/sim.3697 19757444 PMC3472075

[B3] Bauer M. Gerlach H. Vogelmann T. Preissing F. Stiefel J. Adam D. (2020). Mortality in sepsis and septic shock in Europe, North America and Australia between 2009 and 2019- results from a systematic review and meta-analysis. Crit. Care 24, 239. doi: 10.1186/s13054-020-02950-2 32430052 PMC7236499

[B4] Benhamou J. Nieves-Ortega R. Nickel C. H. Lampart A. Kuster T. Balestra G. M. . (2025). Human neutrophil lipocalin, procalcitonin, c-reactive protein, and leucocyte count for prediction of bacterial sepsis in emergency department patients. Scand. J. Trauma Resusc Emerg. Med. 33, 112. doi: 10.1186/s13049-025-01429-9 40598497 PMC12210756

[B5] Certo M. Tsai C. H. Pucino V. Ho P. C. Mauro C. (2021). Lactate modulation of immune responses in inflammatory versus tumour microenvironments. Nat. Rev. Immunol. 21, 151–161. doi: 10.1038/s41577-020-0406-2 32839570

[B6] Chen R. Cao F. Liu T. Wang M. Liu X. Peng H. . (2015a). Investigation on myocardial protective effects of shengmai injection in patients with sepsis. J. North Pharm. 12, 95–96.

[B7] Chen R. Cao F. Liu T. Wang M. Liu X. Peng H. . (2015b). Study on the myocardial protective effects of shengmai injection in patients with sepsis. J. North Pharm. 12, 95–96.

[B8] Chen Y. Hu H. Wang C. Wu J. Zan J. Liu Y. (2025). Epigenetic modulation by lactylation in sepsis: linking metabolism to immune dysfunction. J. Inflamm. Res. 18, 7357–7367. doi: 10.2147/jir.S522081 40497149 PMC12151073

[B9] Chen Y. Siow I. Lee K. S. Dai Q. Xiao X. Garg A. . (2026). Efficacy and safety of bridging intravenous thrombolysis prior to endovascular treatment in patients over 80 years old with acute ischemic stroke. Neuroradiology 68, 1159–1165. doi: 10.1007/s00234-026-03997-8 42068362

[B10] de la Fuente-Nunez C. Cesaro A. Hancock R. E. W. (2023). Antibiotic failure: beyond antimicrobial resistance. Drug Resist. Update 71, 101012. doi: 10.1016/j.drup.2023.101012 37924726 PMC12224857

[B11] Ding L. Shangguan H. Wang X. Liu J. Shi Y. Xu X. . (2025). Extraction and purification of Ophiopogon japonicus polysaccharides, structural characterization, biological activity, safety evaluation, chemical modification and potential applications: a review. Int. J. Biol. Macromol. 300, 140282. doi: 10.1016/j.ijbiomac.2025.140282 39863237

[B12] Gao L. Zheng F. Fu Z. Wang W. (2026). Ginsenoside Rb1-enhanced decellularized extracellular matrix hydrogels ameliorates mitochondrial dysfunction and cellular aging in sepsis-induced acute lung injury. J. Ginseng Res. 50, 100906. doi: 10.1016/j.jgr.2025.10.003 41550598 PMC12805551

[B13] Guo N. Liu Q. Jiang Q. Li Y. Liang J. Zhang Y. (2010). Clinical study on coagulation dysfunction in sepsis based on the theory of correlation between Qi and blood. Chin. Med. Modern Distance Educ. China 8, 145–147.

[B14] Ha Y. Wang X. Huang P. Zhang R. Xu X. Li B. . (2019). Effect of Shengmai Injection on Septic Shock,a systematic review and meta-analyse. J. Emergency Traditional Chin. Med. 28, 1893–1898+1915.

[B15] He S. Ye H. Wang Q. He Y. Liu X. Song J. . (2025). Ginsenoside Rb1 targets to HO-1 to improve sepsis by inhibiting ferroptosis. Free Radic. Biol. Med. 226, 13–28. doi: 10.1016/j.freeradbiomed.2024.11.007 39510452

[B16] Huang D. Chen M. Lin K. (2011). Efficacy observation and clinical experience of Shengmai Injection in the management of septic shock. Jilin Med. J. 32, 3436–3438. doi: 10.1016/b978-1-4160-4252-5.50167-2 38826717

[B17] Huang P. Chen B. Yang X. Chen H. Tang J. Chen R. . (2025). Uncovering the potential of Yiqi Huoxue Jiedu formula: a promising adjunct in sepsis and septic shock management. Front. Cell. Infect. Microbiol. 15, 1655393. doi: 10.3389/fcimb.2025.1655393 41170288 PMC12568585

[B18] Huang Y. Zhao E. Zhao G. Zhuo W. Zhao Y. Wang H. . (2026). H3K18 lactylation-mediated SPHK1-SIRT1 feedback loop accelerates pyroptosis of tubular epithelial cells in sepsis-associated acute kidney injury. Theranostics 16, 4768–4786. doi: 10.7150/thno.122991 41799201 PMC12964219

[B19] Kopustinskiene D. M. Bernatoniene J. (2021). Antioxidant effects of Schisandra chinensis fruits and their active constituents. Antioxidants (Basel) 10. doi: 10.3390/antiox10040620 33919588 PMC8073495

[B20] Lai K. Lin G. Chen C. Xu Y. (2024). Development and validation of a predictive model for acute kidney injury in sepsis patients based on recursive partition analysis. J. Intensive Care Med. 39, 465–476. doi: 10.1177/08850666231214243 37964547

[B21] Luo M. Yan D. Sun Q. Tao J. Xu L. Sun H. . (2020). Ginsenoside Rg1 attenuates cardiomyocyte apoptosis and inflammation via the TLR4/NF-kB/NLRP3 pathway. J. Cell. Biochem. 121, 2994–3004. doi: 10.1002/jcb.29556 31709615

[B22] Marques A. Torre C. Pinto R. Sepodes B. Rocha J. (2023). Treatment advances in sepsis and septic shock: modulating pro- and anti-inflammatory mechanisms. J. Clin. Med. 12. doi: 10.3390/jcm12082892 37109229 PMC10142733

[B23] Müller J. Radej J. Horak J. Karvunidis T. Valesova L. Kriz M. . (2023). Lactate: the fallacy of oversimplification. Biomedicines 11. doi: 10.3390/biomedicines11123192 38137413 PMC10741081

[B24] Oh D. H. Kim M. H. Jeong W. Y. Kim Y. C. Kim E. J. Song J. E. . (2019). Risk factors for mortality in patients with low lactate level and septic shock. J. Microbiol. Immunol. Infect. 52, 418–425. doi: 10.1016/j.jmii.2017.08.009 28923305

[B25] Palakshappa J. A. Taylor S. P. (2025). Management of sepsis in hospitalized patients. Ann. Intern. Med. 178, Itc161–itc176. doi: 10.7326/annals-25-02685 41213152

[B26] Peng X. Zhang J. Lin Y. Li R. (2026). Predicting central lymph node metastasis in papillary thyroid microcarcinoma: a study of ultrasound and clinical features. Front. Endocrinol. (Lausanne) 17, 1752405. doi: 10.3389/fendo.2026.1752405 42039136 PMC13106017

[B27] Rafiq S. Shi C. Ghosal S. Dark P. Felton T. Kontopantelis E. . (2026). Clinical effectiveness of procalcitonin- or C-reactive protein-guided antibiotic discontinuation protocols for adult patients who are critically ill with sepsis: a rapid systematic review and meta-analysis. Anaesthesia 81, 556–569. doi: 10.1111/anae.70109 41505903 PMC12973355

[B28] Rudd K. E. Johnson S. C. Agesa K. M. Shackelford K. A. Tsoi D. Kievlan D. R. . (2020). Global, regional, and national sepsis incidence and mortality 1990-2017: analysis for the Global Burden of Disease Study. Lancet 395, 200–211. doi: 10.1016/s0140-6736(19)32989-7 31954465 PMC6970225

[B29] Shang Y. X. Zheng Z. Wang M. Guo H. X. Chen Y. J. Wu Y. . (2022). Diagnostic performance of Neutrophil CD64 index, procalcitonin, and C-reactive protein for early sepsis in hematological patients. World J. Clin. cases 10, 2127–2137. doi: 10.12998/wjcc.v10.i7.2127 35321184 PMC8895178

[B30] Shi R. Pang S. Xiao L. Wang K. Wang S. Zhang Y. . (2025). Research progress on ginsenosides and their preparations in treatment of sepsis. Chin. Traditional Herbal Drugs 56, 7605–7613.

[B31] Singer M. Angus D. C. Annane D. Bauer M. Kalil A. C. Klompas M. . (2026). Sepsis. Lancet 407, 1276–1288. doi: 10.1016/s0140-6736(25)02422-5 41765030

[B32] Singer M. Deutschman C. S. Seymour C. W. Shankar-Hari M. Annane D. Bauer M. . (2016). The third international consensus definitions for sepsis and septic shock (Sepsis-3). Jama 315, 801–810. doi: 10.1001/jama.2016.0287 26903338 PMC4968574

[B33] Tang Y. Zheng X. Yang X. Guo S. Luo Q. Su M. . (2025). Machine learning-based integration of pericoronary adipose tissue and clinical risk factors for cardiovascular risk prediction in type 2 diabetes: a retrospective cohort study. Eur. J. Med. Res. 30, 961. doi: 10.1186/s40001-025-03237-4 41074084 PMC12512623

[B34] Tchatat Wangueu L. Correia E. Tamion F. Besnier E. (2025). Unlocking the metabolic and anti-inflammatory therapeutic potential of lactate in critically ill patients. Crit. Care 29, 450. doi: 10.1186/s13054-025-05665-4 41137071 PMC12553197

[B35] Wang X. Lin S. Zhong M. Song J. (2024). The procalcitonin trajectory as an effective tool for identifying sepsis patients at high risk of mortality. Crit. Care 28, 312. doi: 10.1186/s13054-024-05100-0 39300542 PMC11411760

[B36] Wang Y. Wei S. Lian H. Tong L. Yang L. Ren B. . (2023). A neutral polysaccharide from spores of Ophiocordyceps gracilis regulates oxidative stress via NRF2/FNIP1 pathway. Int. J. Mol. Sci. 24. doi: 10.3390/ijms241914721 37834168 PMC10572349

[B37] Ward M. A. Kuttab H. I. Badgett R. G. (2025). The effect of early fluid resuscitation on mortality in sepsis: a systematic review and meta-analysis. Crit. Care Med. 53, e1790–e1802. doi: 10.1097/ccm.0000000000006769 40637496

[B38] Wirz Y. Meier M. A. Bouadma L. Luyt C. E. Wolff M. Chastre J. . (2018). Effect of procalcitonin-guided antibiotic treatment on clinical outcomes in intensive care unit patients with infection and sepsis patients: a patient-level meta-analysis of randomized trials. Crit. Care 22, 191. doi: 10.1186/s13054-018-2125-7 30111341 PMC6092799

[B39] Wu J. Li Z. Dong X. Liu J. Wang L. (2025). Shenmai Injection enhances short-term outcomes in ischemic stroke patients after thrombolysis via AMPKα1. Front. Pharmacol. 16, 1552493. doi: 10.3389/fphar.2025.1552493 40376271 PMC12078230

[B40] Xue X. Huang C. Chen J. Ding W. Ren Y. Zhang W. . (2026). Ginsenoside Rg1 mitigates sepsis-associated acute respiratory distress syndrome by promoting autophagy through the Prdx1-PTEN/PI3K/AKT pathway. Phytomedicine 154, 158027. doi: 10.1016/j.phymed.2026.158027 41802381

[B41] Yang C. Ma C. Xu C. S. Li S. R. Li C. Wang Z. F. . (2025). Comprehensive evaluation of frailty and sarcopenia markers to predict survival in glioblastoma patients. J. Cachexia Sarcopenia Muscle 16, e13809. doi: 10.1002/jcsm.13809 40234099 PMC11999731

[B43] Yang L. Wang Y. Ying J. Xiao F. (2021). Effects of Shengmai injection combined with alprostadil on inflammation and oxidative stress in patients with acute liver injury due to sepsis. J. Trop. Med. 21, 637–640+665.

[B42] Yang K. Xu J. Fan M. Tu F. Wang X. Ha T. . (2020). Lactate suppresses macrophage pro-inflammatory response to LPS stimulation by inhibition of YAP and NF-κB activation via GPR81-mediated signaling. Front. Immunol. 11, 587913. doi: 10.3389/fimmu.2020.587913 33123172 PMC7573489

[B44] Yao J. Zhang Q. Xuan J. Cai F. (2021). Clinical study on Shengmai Injection for patients with septic shock in ICU. New Chin. Med. 53, 94–97. doi: 10.13457/j.cnki.jncm.2021.17.023

[B45] Yao Z. W. Zhu H. (2025). Pharmacological mechanisms and drug delivery systems of ginsenoside Rg3: a comprehensive review. Pharmacol. Res. 216, 107799. doi: 10.1016/j.phrs.2025.107799 40414584

[B46] Yu T. Tang Y. Zhang F. Zhang L. (2023). Roles of ginsenosides in sepsis. J. Ginseng Res. 47, 1–8. doi: 10.1016/j.jgr.2022.05.004 36644389 PMC9834008

[B47] Zeng X. Tao H. Dong Y. Zhang Y. Yang J. Xuan F. . (2024). Impact of three-dimensional reconstruction visualization technology on short-term and long-term outcomes after hepatectomy in patients with hepatocellular carcinoma: a propensity-score-matched and inverse probability of treatment-weighted multicenter study. Int. J. Surg. 110, 1663–1676. doi: 10.1097/js9.0000000000001047 38241321 PMC10942183

[B48] Zhang H. Meng F. Tang J. Zhang M. Jing Q. Peng G. . (2025). Lactate inhibits T-cell activation in sepsis through CD40LG downregulation and SOCS3-mediated JAK1/STAT3 pathway suppression. Biochim. Biophys. Acta Mol. Basis Dis. 1871, 167923. doi: 10.1016/j.bbadis.2025.167923 40436284

