# Supplementary material for: Shengmai Injection as adjunctive therapy in sepsis and septic shock: a retrospective cohort study
Source: Front Cell Infect Microbiol. 2026 Jul 30;16:1870034. doi: 10.3389/fcimb.2026.1870034 (PMC13470665; doi:10.3389/fcimb.2026.1870034)
Supplement: Supplementary file 1 [file SupplementaryFile1.docx]

**Supplementary Figures**

**Figure S_1_** Linear regression analyses of variables associated with SMI dosage or medication course with nonsignificant differences in sepsis.

**Figure S_1-1_** Linear regression results for length of ICU stay in sepsis.

**Figure S_1-2_** Linear regression results for duration of invasive ventilator in sepsis.

**Figure S_1-3_** Linear regression results for duration of fever duration in sepsis.

**Figure S_1-4_** Linear regression results for post-treatment lymphocyte in sepsis.

**Figure S_1-5_** Linear regression results for post-treatment neutrophil in sepsis.

**Figure S_1-6_** Linear regression results for post-treatment platelet in sepsis.

**Figure S_1-7_** Linear regression results for post-treatment procalcitonin in sepsis.

**Figure S_2_** Kaplan–Meier survival curves of variables with nonsignificant differences in sepsis.

**Figure S_2-1_** Kaplan–Meier survival curves for age in sepsis.

Age1:SMI group+Age<60 years;Age2:60≤SMI group+Age<75 years;Age3:SMI group+Age≥75 years;Age4:Control group+Age<60 years;Age5:60≤Control group+Age<75 years;Age6:Control group+Age≥75 years.

**Figure S_2-2_** Kaplan–Meier survival curves for drug dosage in sepsis.

Drug1:0<Drug dosage<50ml;Drug2:Drug dosage≥50mL

**Figure S_2-3_** Kaplan–Meier survival curves for grouping in sepsis.

**Figure S_2-4_** Kaplan–Meier survival curves for post-treatment lactate in sepsis.

Lactate 1:SMI group+ Lactate<2mmol/L; Lactate 2:SMI group+ Lactate≥2mmol/L; Lactate 3: Control group+ Lactate<2mmol/L; Lactate 4: Control group+ Lactate≥2mmol/L.

**Figure S_2-5_** Kaplan–Meier survival curves for post-treatment SOFA scores in sepsis.

SOFA1:SMI group+Post-treatment SOFA scores<2 scores;SOFA2:SMI group+Post-treatment SOFA scores≥2 scores;SOFA3:Control group+Post-treatment SOFA scores<2 scores;SOFA4:Control group+Post-treatment SOFA scores≥2 scores

**Figure S_3_** Linear regression analyses of variables associated with SMI dosage or medication course with nonsignificant differences in septic shock.

**Figure S_3-1_** Linear regression results for duration of invasive ventilator in septic shock.

**Figure S_3-2_** Linear regression results for post-treatment SOFA scores in septic shock.

**Figure S_3-3_** Linear regression results for post-treatment APACHE II scores in septic shock.

**Figure S_3-4_** Linear regression results for post-treatment platelet in septic shock.

**Figure S_4_** Kaplan–Meier survival curves of variables with nonsignificant differences in septic shock.

**Figure S_4-1_** Kaplan–Meier survival curves for age in septic shock.

Age1:SMI group+Age<60 years;Age2:60≤SMI group+Age<75 years;Age3:SMI group+Age≥75 years;Age4:Control group+Age<60 years;Age5:60≤Control group+Age<75 years;Age6:Control group+Age≥75 years.

**Figure S_4-2_** Kaplan–Meier survival curves for drug dosage in septic shock.

Drug1:0<Drug dosage<50ml;Drug2:Drug dosage≥50mL

**Figure S_4-3_** Kaplan–Meier survival curves for grouping in septic shock.

**Figure S_4-4_** Kaplan–Meier survival curves for post-treatment medication course in septic shock.

Course 1: 0<Medication course<7 days; Course 2: Medication course≥7 days.

**Figure S_4-5_** Kaplan–Meier survival curves for post-treatment SOFA scores in septic shock.

SOFA1:SMI group+Post-treatment SOFA scores<2 scores;SOFA2:SMI group+Post-treatment SOFA scores≥2 scores;SOFA3:Control group+Post-treatment SOFA scores<2 scores;SOFA4:Control group+Post-treatment SOFA scores≥2 scores

**Figure S_4-6_** Kaplan–Meier survival curves for post-treatment procalcitonin in septic shock.

Procalcitonin 1:SMI group + PCT < 2 ng/mL; Procalcitonin 2:SMI group + PCT ≥ 2 ng/mL; Procalcitonin 3: Control group + PCT < 2 ng/mL; Procalcitonin 4: Control group + PCT ≥ 2 ng/mL .

**Figure S_4-7_** Kaplan–Meier survival curves for post-treatment lactate in septic shock.

Lactate 1:SMI group+ Lactate<2mmol/L; Lactate 2:SMI group+ Lactate≥2mmol/L; Lactate 3: Control group+ Lactate<2mmol/L; Lactate 4: Control group+ Lactate≥2mmol/L.

**Note:**Despite the highly significant log-rank test result (*p* < 0.001), we were unable to establish post-treatment lactate as a prognostic factor for 28-day mortality in septic shock, due to the absence of Lactate 2 measurements.

Figure 5.The post-hoc power analysis and sample size calculation of sepsis.

**Figure S_5-1_** The post-hoc power analysis of sepsis.

*w* = 0.0992, a=0.05,df=1,power = 0.5214

**Figure S_5-2_** The sample size calculation of sepsis.

*w* = 0.0992, a=0.05,df=1,power =0.80，n=798
