# Supplementary material for: Shengmai Injection as adjunctive therapy in sepsis and septic shock: a retrospective cohort study
Source: Front Cell Infect Microbiol. 2026 Jul 30;16:1870034. doi: 10.3389/fcimb.2026.1870034 (PMC13470665; doi:10.3389/fcimb.2026.1870034)
Supplement: Supplementary file 2 [file Table1.docx]

**Supplementary Table 1.** **Chemical components identified in SMI using UPLC-MS analysis.**

| **RT（min）** | **Formula** | **Compound CID** | **Pseudomolecular ion（m/z）** | **Error(ppm)** | **MS/MS fragments** | **Molecule Name** |
| --- | --- | --- | --- | --- | --- | --- |
| 1.753 | C_6_H_6_O_3_ | 17756553 | [M+H]+:127.0404 | - | 109.0303,111.0456 | 3-Hydroxymethyl-2-furfural |
| 4.260 | C_23_H_26_O_7_ | 131873798 | [M + Na]+:437.1611 | 9.15 | 366.1049,347.0944,305.0838,  275.1097 | schisanlignone C |
| 4.440 | C_7_H_6_O_4_ | 72 | [M − H]−:153.0188 | -3.40 | 137.0112,108.0449,93.0216 | 3, 4-dihydroxybenzoic acid |
| 4.520 | C_7_H_10_O_5_ | 8742 | [M + COOH]−:219.0498 | -5.48 | - | shikimic acid |
| 4.890 | C_7_H_10_O_7_ | 12566215 | [M − H]−:205.0349 | 2.37 | - | Citric acid mono-methyl ester |
| 5.445 | C_12_H_16_O_8_ | 5316639 | [M + COOH]−:333.0835 | -2.70 | 287.1009,125.0244 | Dianthoside |
| 6.165 | C_33_H_44_O_3_ | 145709631 | [M+H]+:453.3445，[M+Na]+:475.3260 | - | 435.3328,339.2723,227.1785 | Neokadsuranic acid B |
| 6.311 | C_42_H_72_O_15_ | 177825113 | [M + COOH]−:861.4831,  [M − H]−:815.4774 | -2.30 | 653.2431，553.3397，521.2028，391.1734 | Majonoside R_1_/Momorcharassikde A |
| 6.567 | C_48_H_82_O_19_ | 24721561 | [M + COOH]−:1007.5421,  [M − H]−:961.5358 | -1.40 | 799.4791，781.4736，637.4299，619.2382,475.1818 | Notoginsenoside N/M/R_6_/20-glc-ginsenoside Rf |
| 6.567 | C_42_H_74_O_15_ | 131752636 | [M + COOH]−:863.4997,  [M − H]−:817.4937 | -1.60 | 655.4421,493.3854 | Quinquenoside F_1_ |
| 7.038 | C_6_H_6_O_3_ | 237332 | [M + H]+:127.0391 | -0.89 | 109.0283 | 5-Hydroxymethyl furfural |
| 9.404 | C_53_H_90_O_22_ | 12855920 | [M + COOH]−:1123.5892,  [M − H]−:1077.5836 | -0.80 | 475.3794 | Ginsenoside Rha |
| 10.649 | C_21_H_36_O_10_ | - | [M + COOH]−:493.2288,  [M − H]−:- 447.2232 | 0.40 | 315.1813 | L-borneol-7-O-[β-D-apiofuranosyl(1→6)]  -β-D-glucopy-ranoside |
| 11.570 | C_44_H_70_O_16_ | 46173859 | [M − H]−:853.4526 | -7.64 | 835.4526, 689.4526, 557.4526 | ophiopogonin D |
| 11.790 | C_21_H_20_O_11_ | 5280459 | [M − H]−:447.0909 | -5.52 | 401.0909 | quercitrin |
| 11.930 | C_29_H_28_O_8_ | 21125391 | [M + Na]+：527.1716 | 7.59 | - | interiotherin A |
| 12.000 | C_44_H_70_O_18_ | 102004869 | [M − H]−:885.4476 | -1.49 | 753.4476, 607.4476, 445.4476 | cixi-ophiopogon A |
| 12.278 | C_41_H_70_O_23_ | 21599925 | [M + COOH]−: 815.4780,  [M − H]−:769.4725 | -1.70 | 637.4309,619.4201,475.3795 | Notoginsenoside R_2_/F_3_/F_5_ |
| 12.278 | C_44_H_74_O_15_ | 11136628 | [M + COOH]−: 887.4987 ,  [M − H]−:841.4935 | -1.70 | 841.4935,799.4832,781.4727,  637.4310,619.4202,475.3791 | Ace-ginsenoside Rg_1_ |
| 12.466 | C_44_H_74_O_15_ | 44584744 | [M + COOH]−: 887.4986 ,  [M − H]−:841.4934 | -1.80 | 841.4933,799.4829,781.4726,  637.3069,619.4210,475.3783 | Ace-ginsenoside Rf |
| 12.913 | C_30_H_46_O_4_ | 10814237 | [M + H]+:471.3459 | - | 133.1021,145.0508,159.1171,  173.1330 | Nigranoic acid |
| 13.040 | C_47_H_80_O_17_ | 131751558 | [M + COOH]−:961.5361 | -1.77 | 915.5312, 783.4898, 475.3771 | vinaginsenoside R_16_ |
| 13.082 | C_51_H_86_O_21_ | 85212395 | [M + COOH]−: 1033.5549,  [M − H]−:987.5510 | -1.90 | 945.5407，783.4875，765.4809，621.2800，459.1711 | Quinquenoside III |
| 13.703 | C_30_H_46_O_3_ | 5288149 | [M + H]+:455.3528 | - | 437.3410,187.1495,175.1491,  161.1341 | Glucosinic acid |
| 14.157 | C_59_H_100_O_27_ | 177824497 | [M + COOH]−:1285.6155 ,  [M − H]−:1239.6361 | -1.00 | 1107.5936,1077.5874,945.5415,783.4885,765.4800,621.4368,  459.3844 | Notoginsenoside R_4_/Ra_3_/Fa |
| 14.392 | C_63_H_106_O_30_ | 21674164 | [M − H]−:1341.6648 | -1.50 | 1209.6302,1077.5874,1047.5726,945.5390,783.4884,765.4772,621.4347,459.3809 | Notoginsenoside Q |
| 14.797 | C_41_H_70_O_13_ | 21599925 | [M + COOH]−: 815.4790 ,  [M − H]−:769.4733 | -0.60 | 637.4318,619.4218,475.3790 | Notoginsenoside R_2_/F_3_/F_5_ |
| 14.844 | C_59_H_100_O_27_ | 44592854 | [M − H]−:1239.6331 | -3.50 | 1107.5883,1077.5829,945.5391 | Notoginsenoside R_4_/Ra_3_/Fa |
| 14.910 | C_15_H_10_O_7_ | 5280343 | [M + H]+:303.0488 | -3.73 | 301.0488 | quercetin |
| 14.950 | C_29_H_34_O_7_ | 14992069 | [M + Na]+:517.2236 | 7.54 | - | Benzoylgomisin H |
| 15.573 | C_54_H_90_O_23_ | 131751883 | [M − H]−:1105.5775 | -1.70 | 943.5251,781.4737,763.4636,  619.4199,457.3692 | Quadrangulcoside |
| 15.880 | C_25_H_30_O_8_ | 171064 | [M + H]+:459.1972 | -8.99 | - | kadsurin |
| 15.972 | C_58_H_98_O_26_ | 75412556 | [M − H]−:1209.6211 | 0.20 | 1077.5820,1047.5768,945.5323,915.5342,783.4874,765.4772,  621.4296,603.4311,459.3806 | Notoginsenoside Fc/Ra_1_/Ra_2_ |
| 17.037 | C_51_H_84_O_21_ | 14162967 | [M + COOH]−:1077.5782 ,  [M − H]−:1031.5449 | 2.10 | 945.5397,783.4885,765.4738,  621.4348,459.3800 | Mal-ginsenoside Rd |
| 17.346 | C_30_H_48_O_3_ | 10494 | [M + H]+:457.3658 | 3.97 | 439.3557 | Oleanolic acid |
| 17.350 | C_27_H_32_O_7_ | 358883 | [M + H]+:469.2208 | -2.62 | 459.7354,437.2364 | angeloyl binankadsurin A |
| 17.528 | C_57_H_94_O_26_ | 118987129 | [M + COOH]−:1239.6329 ,  [M − H]−:1193.5938 | -1.60 | 1149.6061,1107.5950,1089.5836,945.5453,927.5250,783.4896,765.4796,621.4360,459.3821 | Mal-ginsenoside Rb_1_ |
| 17.748 | C_56_H_94_O_24_ | 131752965 | [M + COOH]−: 1195.6368,  [M − H]−:1149.6046 | -1.00 | 1107.5947,1089.5837,945.5456,927.5250,783.4906,765.4791,  621.4381,459.3821 | Ace-ginsenoside Rb_1_/Quinquenoside R_1_ |
| 17.748 | C_48_H_76_O_19_ | 13919548 | [M − H]−:955.4908 | 0.50 | 731.4353,613.3730,587.4069,  569.3745,523.3809,455.3567 | Tuberoside A/Amaramthussaponin |
| 18.530 | C_23_H_28_O_6_ | 158103 | [M + H]+:401.1968 | 2.42 | 369.1697,337.1436,309.1486 | gomisin N |
| 18.688 | C_58_H_98_O_26_ | 75412556 | [M + COOH]−:1255.5912 ,  [M − H]−:1209.6252 | -1.10 | 1077.5836,1047.5646,945.5413,915.5310,783.4894,765.4786,  621.4372,603.4270,459.3852 | Notoginsenoside Fc/Ra_1_/Ra_2_ |
| 18.780 | C_39_H_62_O_12_ | 46173857 | [M + H]+:723.4348 | 4.73 | 251.4345 | Ophiopogonin B |
| 19.660 | C_23_H_30_O_6_ | 73057 | [M + H]+:403.2119 | 1.17 | 302.1090, 271.0882 | Schisanhenol /Gomisin K_1_/GomisinK_2_ |
| 20.289 | C_47_H_74_O_18_ | 11766338 | [M + COOH]−: 971.5169,  [M − H]−:925.4792 | -0.50 | 763.4269,613.3699,587.4039,  569.3914,455.3524 | Stipuleanoside R_1_ |
| 20.660 | C_43_H_68_O_14_ | 637855 | [M − H]−:807.4510 | -3.24 | - | Chikusetsusaponin IVa methyl ester |
| 21.160 | C_45_H_74_O_19_ | 21422914 | [M + H]+:919.4948 | 5.57 | - | ophiofurospiside J |
| 22.683 | C_24_H_32_O_7_ | 3001664 | [M + H]+:433.2212 | - | - | Schisandrin/Gomisin S/Isoschizandrin  /Schizandrol A |
| 22.766 | C_60_H_100_O_27_ | 177827773 | [M − H]−:1251.6343 | -3.80 | 1209.6238,1077.5815,1059.5759,945.5365,915.3580,783.4876,765.4770,621.4375,459.3867 | Ace-ginsenoside Ra_1_/Ra_2_ |
| 26.180 | C_48_H_82_O_18_ | 11679800 | [M + COOH]−:991.5458 ,  [M − H]−:945.5404 | -2.00 | 783.4869,765.4753,621.4350,  603.4334,459.3888 | Ginsenoside Rd isomer |
| 27.227 | C_42_H_70_O_13_ | 102179725 | [M + COOH]−: 827.4732,  [M − H]−:781.4719 | -2.40 | 619.4207 | Ginsenoside Rg_8_/Rg_9_ |
| 29.520 | C_42_H_72_O_13_ | 9918693 | [M − H]−:783.4856 | -5.64 | 621.4269，537.3412，459.3824，375.2885 | ginsenoside Rg_3_ |
| 31.413 | C_42_H_66_O_14_ | 13909684 | [M − H]−:793.4387 | 1.60 | 731.4388,631.3867,613.3751,  587.4146,569.3860,537.3511,  523.3809,455.3523 | Chikusetsu saponin IVa |
| 31.755 | C_42_H_72_O_13_ | 9918693 | [M + COOH]−: 829.4942 ,  [M − H]−:783.4879 | -2.00 | 621.4362,459.3850 | Ginsenoside Rg_3_/Fc/F_2_ |
| 32.280 | C_58_H_98_O_26_ | 100941542 | [M − H]−:1209.6199 | -6.13 | 1077.5836,945.5408，915.5301，783.4877,621.4353 ,459.3829 | ginsengoside Ra_1_ |
| 32.700 | C_26_H_30_O_8_ | 145709337 | [M + H]+:471.1971 | -8.98 | - | Kadsulignan H |
| 32.720 | C_59_H_100_O_27_ | 75054973 | [M + COOH]−:1285.6350 | -6.53 | 1249.5217,1107.5900，1078.6128,922.4577 | notoginsenoside Fa |
| 33.890 | C_23_H_30_O_7_ | 14213996 | [M + Na]+:441.1866 | -4.08 | 423.1886 | gomisin U |
| 34.610 | C_57_H_93_O_23_ | - | [M + COOH]−:1190.6110 | 1.68 | - | ginsengoside Ra_7_ |
| 35.775 | C_44_H_74_O_14_ | 100937823 | [M + COOH]−:871.5005 ,  [M − H]−:825.4982 | -2.20 | 783.4879,621.4351,459.3838 | Ginsenoside Rs_3_ |
| 36.454 | C_10_H_16_O | 638011 | [M + H]+:153.1267 | 4.52 | 107.0855 | Citral |
| 39.290 | C_48_H_82_O_18_ | 11679800 | [M − H]−:945.5357 | -7.51 | 783.4878,637.4303,475.3778 | ginsenoside Rd |
| 39.626 | C_22_H_26_O_6_ | 5321170 | [M + H]+:387.1793 | 2.25 | 372.1547,355.1525,345.1332,  331.1173,299.0886 | Gomisin M_1_/Gomisin M_2_/Gomisin L_1_/Gomisin L_2_ |
| 39.850 | C_55_H_92_O_23_ | 163106632 | [M + COOH]−:1165.5968 | -3.69 | 1119.5908,1047.5691,946.5538,625.3251 | ginsengoside Rs_1_/Rs_2_ |
| 40.570 | C_27_H_34_O_8_ | 5120984 | [M + Na]+:509.2147 | 0.20 | - | isovaleroyl binankadsurin A |
| 40.815 | C_21_H_30_O_11_ | 71312510 | [M + Na]+:481.1695 | -3.09 | - | 4-allyl-1,2-benzenediol 1-O-α-L-rhamnpyranosyl  (1→6)-O-β-D-glu-copyrano-side |
| 41.910 | C_18_H_18_O_5_ | 14826840 | [M + H]+:315.1220 | -2.16 | 192.0406 | ophiopogonone B |
| 41.910 | C_19_H_18_O_5_ | 23259413 | [M + H]+:327.1220 | -2.08 | 206.0578, 178.0633 | methylophiopogonone B |
| 41.910 | C_19_H_20_O_5_ | 46886723 | [M + H]+:329.1379 | -1.46 | - | methylophiopogonanone B |
| 41.918 | C_48_H_82_O_19_ | 24721561 | [M − H]−:961.5397 | -2.07 | 799.4859,637.4410,475.3777 | 20-glc-Ginsenoside Rf |
| 41.997 | C_47_H_80_O_17_ | 91973814 | [M + COOH]−:961.5407 | -3.21 | - | Notoginsenoside Ft_1_ |
| 42.151 | C_44_H_72_O_13_ | 163020413 | [M + COOH]−:853.4960,  [M − H]−:807.4899 | 0.50 | 765.4799,747.4692,603.4272,  537.3942,441.2397 | Ginsenoside Rs4/ginsenoside Rs5/  Ace-ginsenoside Rg5/Ace-ginsenoside Rk1 |
| 42.869 | C_47_H_80_O_18_ | 441934 | [M − H]−:931.5313 | -4.38 | 799.4845,637.4368,475.3841 | Notoginsenoside R_1_ |
| 43.780 | C_27_H_32_O_8_ | 145709656 | [M + H]+:485.2150 | -3.98 | - | kadsulignan J |
| 43.780 | C_19_H_16_O_6_ | 53468060 | [M + H]+:341.1006 | -4.05 | - | 8-formylophiopogonone B |
| 44.600 | C_42_H_66_O_14_ | 13909684 | [M − H]−:793.4333 | -5.95 | 569.3821，479.3872 | [Chikusetsusaponin Iva](https://www.chemsrc.com/en/cas/51415-02-2_1170588.html) |
| 44.693 | C_42_H_72_O_14_ | 441923 | [M + COOH]−:845.4954,  [M − H]−:799.4844 | -6.21 | 637.4335,475.2823 | Ginsenoside Rg_1_ |
| 44.931 | C_48_H_82_O_18_ | 441921 | [M − H]−:945.5456 | -3.58 | 799.4867,783.4902,637.4381,  475.3824 | Ginsenoside Re |
| 45.620 | C_42_H_72_O_13_ | 21599924 | [M + COOH]−:829.4911 | -5.30 | 783.5239,621.4357,459.3828 | ginsenoside Rg_2_ |
| 46.048 | C_10_H_20_O | 8842 | [M + H]+:157.1580 | 4.29 | - | Citronellol |
| 46.270 | C_22_H_24_O_6_ | 443027 | [M + H]+:385.1633 | -3.19 | 367.1630, 321.1629 | Schisandrin C |
| 47.920 | C_44_H_74_O_14_ | 100937823 | [M − H]−:825.4971 | -4.26 | 785.4922 | ginsengoside Rs |
| 48.170 | C_24_H_30_O_7_ | 14827754 | [M + Na]+:453.1868 | -3.53 | 422.1884 | schisanlignone A |
| 48.170 | C_17_H_14_O_5_ | 129848217 | [M + H]+:299.0904 | -3.28 | - | desmethylisoophiopogonone B |
| 48.220 | C_15_H_10_O_5_ | 5280443 | [M + COOH]−:315.0497 | -4.13 | 297.0495 | Apigenin |
| 50.150 | C_16_H_32_O_2_ | 985 | [M − H]−:255.2308 | -8.50 | - | Palmitic acid |
| 55.793 | C_60_H_102_O_28_ | 102601548 | [M − H]−:1269.6442 | 3.10 | - | Ginsenoside Ra_0_/notoginsenoside R_4_ |
| 56.189 | C_42_H_72_O_14_ | 441922 | [M − H]−:799.4849 | -0.11 | 637.4270,475.3787 | Ginsenoside Rf |
| 57.141 | C_58_H_98_O_26_ | 100941542 | [M − H]−:1209.6277 | -0.26 | 1077.5848,945.5364,783.4867,  621.4326 | Ginsenoside Ra_1_/Ginsenoside Ra_2_ |
| 57.537 | C_59_H_100_O_27_ | 73157064 | [M − H]−:1239.6345 | 0.59 | 1107.5958,945.5429,783.4661 | Ginsenoside Ra_3_ |
| 57.617 | C_54_H_92_O_23_ | 9898279 | [M − H]−:1107.5966 | -0.62 | 945.5413,783.4933 | Ginsenoside Rb_1_ |
| 58.013 | C_41_H_70_O_13_ | 21599925 | [M − H]−:769.4765 | -2.63 | 637.4291,475.3815 | Notoginsenoside R_2_ |
| 58.489 | C_57_H_94_O_26_ | 101835367 | [M − H]−:1193.5963 | -0.18 | 1149.6044 | Manoyl-Ginsenoside Rb_1_/isomer |
| 58.965 | C_53_H_90_O_22_ | 12855889 | [M − H]−:1077.5861 | -1.49 | 945.5451,915.5321,783.4949,  621.4356,459.3836 | Ginsenoside Rc |
| 59.678 | C_42_H_72_O_13_ | 21599924 | [M + COOH]−:829.4987,  [M − H]−:783.4915 | -4.03 | 637.4329,475.3812 | S-Ginsenoside Rg_2_ |
| 59.916 | C_42_H_72_O_13_ | 12912318 | [M + COOH]−:829.4972,  [M − H]−:783.4892 | -2.19 | 637.4338,475.3787 | R-Ginsenoside Rg_2_ |
| 60.154 | C_36_H_62_O_9_ | 12855920 | [M + COOH]−:683.4403 | -4.25 | 637.4251,475.3854 | Ginsenoside Rh_1_ |
| 60.392 | C_48_H_76_O_19_ | 11815492 | [M − H]−:955.4920 | -3.16 | 793.4384,631.3796,455.3572 | Ginsenoside Ro |
| 60.471 | C_53_H_90_O_22_ | 6917976 | [M − H]−:1077.5870 | -1.61 | 945.5444,915.5215,783.4925,  621.4418 | Ginsenoside Rb_2_ |
| 61.026 | C_53_H_90_O_22_ | 12912363 | [M − H]−:1077.5876 | -2.09 | 945.5521,915.5379,783.4883,  765.4774,621.4410,459.3729 | Ginsenoside Rb_3_ |
| 61.423 | C_56_H_92_O_25_ | 101835368 | [M − H]−:1163.5870 | -1.42 | 1119.5952 | Manoyl-Ginsenoside Rc/Rb_2_/Rb_3_/isomer |
| 63.088 | C_47_H_74_O_18_ | 15602013 | [M − H]−:925.4828 | -2.52 | 775.4251,613.3770,569.3840,  492.4867 | Chikusetsu saponin IV |
| 64.515 | C_36_H_62_O_9_ | 9809542 | [M + COOH]−:683.4385 | -1.43 | 637.4355,475.3751 | Ginsenoside F_1_ |
| 65.228 | C_48_H_82_O_18_ | 11679800 | [M − H]−:945.5432 | -0.44 | 783.4893,621.4377,459.3738,  375.2837 | Ginsenoside Rd |
| 66.267 | C_23_H_30_O_7_ | 102004650 | [M + H]+:419.2052 | 2.97 | 401.1978,370.1679 | Gomisin H/Gomisin T |
| 66.565 | C_42_H_66_O_14_ | 13909684 | [M − H]−:793.4385 | -0.62 | 631.3846 | Chikusetsu saponin IVa |
| 67.607 | C_55_H_92_O_23_ | 85044013 | [M − H]−:1119.5935 | 1.84 | 1077.5899,1059.5684,945.5240,915.5232,783.4881,765.4685 | Ginsenoside R_S1_/R_S2_ |
| 72.927 | C_24_H_32_O_7_ | 14213995 | [M + H]+:433.2207 | 3.09 | 415.2111,400.1856,384.1914 | Gomisin S |
| 79.270 | C_24_H_32_O_7_ | 10455507 | [M + H]+:433.2205 | 3.68 | 415.2104,400.1880,384.1928 | Isoschizandrin |
| 79.580 | C_42_H_70_O_12_ | 91895489 | [M + COOH]−:811.4858 | -1.09 | 765.4738,619.4192,601.4118 | Ginsenoside Rg_6_ |
| 80.829 | C_24_H_32_O_7_ | 3001664 | [M + H]+:433.2160 | -4.40 | 415.2104,384.1920 | Schizandrol A |
| 81.403 | C_42_H_70_O_12_ | 73717457 | [M + COOH]−:811.4851 | -0.17 | 765.4796,619.4216,601.4278 | Ginsenoside F_4_ |
| 82.593 | C_36_H_60_O_8_ | 21599928 | [M + COOH]−:665.4270 | 0.05 | 505.1742,390.7307 | Ginsenoside Rh_4_/Rk_3_ |
| 85.526 | C_42_H_66_O_14_ | 10395524 | [M − H]−:799.4366 | 1.82 | 613.3712 | Zingibroside R_1_ |
| 86.961 | C_28_H_34_O_10_ | 5317799 | [M + NH4]+:548.2445,  [M + H]+:531.2189 | 2.00 | 485.2145,401.1580 | Gomisin D |
| 88.460 | C_42_H_72_O_13_ | 9918693 | [M − H]−:783.4888 | 1.66 | 621.4398 | S-Ginsenoside Rg_3_ |
| 88.618 | C_42_H_72_O_13_ | 46887680 | [M − H]−:783.4887 | 1.79 | 621.4371,459.3773 | R-Ginsenoside Rg_3_ |
| 89.578 | C_22_H_28_O_6_ | 3001686 | [M + H]+:389.1980 | 0.43 | 374.1535,358.1687,319.1155 | Gomisin J |
| 90.212 | C_23_H_28_O_7_ | 3001662 | [M + H]+:417.1892 | -1.52 | - | Schizandrol B |
| 90.442 | C_42_H_72_O_13_ | 9918692 | [M + COOH]−:829.4952 | 0.36 | 783.4877,621.4375,459.3870 | Ginsenoside F_2_ |
| 91.639 | C_32_H_50_O_4_ | 5318776 | [M + Na]+:521.3598 | 0.66 | 423.1389,379.2213,361.2146,  343.1967 | Tigloylgomisin O |
| 94.251 | C_28_H_34_O_9_ | 51003489 | [M + H]+:515.2251 | 4.62 | 385.1624 | Gomisin F/Tigloylgomisin P |
| 94.731 | C_28_H_34_O_9_ | 78385000 | [M + H]+:515.2253 | 4.44 | 385.1624,355.1523 | Gomisin E/Schizantherin C |
| 96.555 | C_28_H_36_O_8_ | 5318766 | [M + H]+:501.2503 | 3.49 | 483.2327,401.1945 | Tigloylgomisin H/Angeloylgomisin H |
| 99.640 | C_44_H_74_O_14_ | 100937823 | [M + COOH]−:871.5031 | -1.43 | 825.5056,783.4868,765.4704,  621.4308,459.3823 | Ginsenoside Rs_3_/isomer |
| 100.916 | C_23_H_28_O_6_ | 158103 | [M + H]+:401.1943 | 3.92 | 386, 1634,370.1805,355.1487 | g-schizandrin |
| 100.995 | C_28_H_36_O_8_ | 26204131 | [M + H]+:501.2461 | 4.39 | 483.2360,401.1928 | Angeloylgomisin H/Tigloylgomisin H |
| 105.348 | C_42_H_70_O_12_ | 11499198 | [M + COOH]−:811.4840 | 1.28 | 765.4753,603.4293 | Ginsenoside Rk_1_ |
| 106.379 | C_42_H_70_O_12_ | 11550001 | [M + COOH]−:811.4841 | 0.94 | 765.4764,603.4232 | Ginsenoside Rg_5_ |
| 110.432 | C_28_H_34_O_9_ | 71307574 | [M + NH4]+:532.2520 | 4.22 | 415.1726,385.1640 | Schizantherin B |
| 118.189 | C_24_H_32_O_6_ | 155256 | [M + H]+:417.2283 | 3.57 | 402.2026,386.2076,347.1481 | Schizandrin A |
