# Supplementary material for: Shengmai Injection as adjunctive therapy in sepsis and septic shock: a retrospective cohort study
Source: Front Cell Infect Microbiol. 2026 Jul 30;16:1870034. doi: 10.3389/fcimb.2026.1870034 (PMC13470665; doi:10.3389/fcimb.2026.1870034)
Supplement: Supplementary file 3 [file Table2.docx]

**Supplementary Table 2. SMD of the SMI and control groups before and after PSM in the Sepsis and Septic shock.**

|  | **Before propensity score matching** | | **After propensity score matching** | |
| --- | --- | --- | --- | --- |
|  | **Sepsis** | **Septic shock** | **Sepsis** | **Septic shock** |
| **Characteristics** | SMD | SMD | SMD | SMD |
| Sex（Men） | 0.050 | 0.098 | 0.029 | 0.094 |
| Age | 0.050 | 0.023 | 0.040 | 0.015 |
| Body mass index | 0.036 | 0.028 | 0.030 | 0.057 |
| **vital signs** |  |  |  |  |
| Body temperature | 0.203 | 0.103 | 0.169 | 0.074 |
| Respiratory rate | 0.146 | 0.214 | 0.098 | 0.301 |
| Heart rate | 0.190 | 0.285 | 0.163 | 0.207 |
| Systolic blood pressure | 0.101 | 0.190 | 0.114 | 0.220 |
| Diastolic pressure | 0.032 | 0.014 | 0.014 | 0.031 |
| SPO_2_ | 0.036 | 0.024 | 0.042 | 0.029 |
| Pre-treatment SOFA scores | 0.086 | 0.402 | 0.058 | 0.440 |
| Pre-treatment APACHE II scores | 0.210 | 0.228 | 0.198 | 0.221 |
| **Source of infection** |  |  |  |  |
| Lung infection | 0.042 | 0.080 | 0.034 | 0.038 |
| Urinary tract infection | 0.185 | 0.277 | 0.188 | 0.223 |
| Skin and soft tissue infection | 0.124 | 0.254 | 0.113 | 0.254 |
| Intra-abdominal infection | 0.101 | 0.056 | 0.069 | <0.001 |
| Bloodstream infection | 0.046 | 0.178 | 0.026 | 0.178 |
| Gastrointestinal infection | 0.083 | 0.255 | 0.074 | 0.260 |
| Mixed infection | 0.153 | 0.094 | 0.155 | 0.069 |
| Other infections | 0.046 | 0.097 | 0.026 | 0.129 |
| **Microorganisms** |  |  |  |  |
| Blood culture | 0.075 | 0.198 | 0.010 | 0.254 |
| Gram-negative bacteria | 0.039 | 0.029 | 0.077 | 0.036 |
| Gram-positive bacteria | 0.027 | 0.090 | 0.044 | 0.138 |
| Fungus | 0.096 | 0.096 | 0.059 | 0.038 |
| Cross-infection | 0.009 | 0.090 | 0.020 | 0.061 |
| Sterile | 0.144 | 0.118 | 0.049 | 0.063 |
| **Anti-infective drugs** |  |  |  |  |
| Antibiotics | 0.064 | 0.168 | 0.057 | <0.001 |
| Antifungal drugs | 0.087 | 0.042 | 0.077 | 0.034 |
| Antiviral drugs | 0.152 | 0.258 | 0.150 | 0.309 |
| Multiple drugs | 0.025 | 0.112 | 0.042 | 0.067 |
| No Anti-infective drugs | 0.064 | 0.168 | 0.057 | <0.001 |
| **Underlying comorbidities** |  |  |  |  |
| Hypertension | 0.049 | 0.193 | 0.030 | 0.158 |
| Coronary heart disease | 0.228 | 0.004 | 0.174 | <0.001 |
| Diabetes | 0.182 | 0.184 | 0.162 | 0.105 |
| Stroke | 0.100 | 0.096 | 0.124 | 0.038 |
| Chronic obstructive pulmonary disease | 0.066 | 0.208 | 0.094 | 0.156 |
| Liver disease | 0.018 | 0.208 | 0.011 | 0.198 |
| Kidney disease | 0.073 | 0.079 | 0.040 | 0.137 |
| Tumor | 0.135 | 0.094 | 0.145 | 0.148 |
| Other diseases | 0.087 | 0.307 | 0.066 | 0.292 |
| **Laboratory results** |  |  |  |  |
| Pre-treatment Procalcitonin | 0.026 | 0.033 | 0.019 | 0.009 |
| Pre-treatment White blood cell | 0.169 | 0.222 | 0.170 | 0.156 |
| Pre-treatment Lymphocyte | 0.170 | 0.228 | 0.167 | 0.243 |
| Pre-treatment Neutrophil | 0.145 | 0.322 | 0.126 | 0.248 |
| Pre-treatment Platelet | 0.060 | 0.083 | 0.083 | 0.044 |
| Pre-treatment NLR | 0.157 | 0.242 | 0.134 | 0.133 |
| Pre-treatment Lactate | 0.133 | 0.150 | 0.126 | 0.165 |

SMI, Shengmai Injection; PSM, Propensity score matching; SPO_2_, Peripheral Capillary Oxygen Saturation; SOFA, Sequential Organ Failure Assessment; APACHE, Acute Physiology and Chronic Health Evaluation; NLR, Neutrophil-to-lymphocyte Ratio.SMD,Standardized Mean Difference.

SMD was used to assess covariate balance, where an SMD <0.1 indicated optimal balance, an SMD between 0.1 and 0.2 indicated mild residual imbalance, and an SMD >0.2 indicated unacceptable imbalance.

We initially intended to perform PSM for the septic shock subgroup using the same 1:1 optimal non-replacement matching strategy adopted for the overall sepsis cohort. However, the septic shock cohort still showed some variable imbalances with SMD > 0.2 even after adopting multiple matching strategies. Given methodological considerations regarding over-matching, PSM was not conducted for septic shock.
