# Supplementary material for: Shengmai Injection as adjunctive therapy in sepsis and septic shock: a retrospective cohort study
Source: Front Cell Infect Microbiol. 2026 Jul 30;16:1870034. doi: 10.3389/fcimb.2026.1870034 (PMC13470665; doi:10.3389/fcimb.2026.1870034)
Supplement: Supplementary file 4 [file Table3.docx]

**Supplementary Table 3.Rosenbaum Bounds sensitivity analysis of sepsis.**

# **Table 3.1 McNemar test（*Γ* = 1.0）**

| statistical magnitude | Pairing of logarithms |
| --- | --- |
| D_1_ | 24 |
| D_0_ | 36 |
| *P* value | 0.155 |

D_1_: Death occurred in patients with SMI, while control patients remained alive.

D_0_: Patients with SMI were alive at endpoint, whereas control patients experienced death.

Under the assumption of no unmeasured confounding (*Γ* = 1.0), the McNemar test produced a P value of 0.155, which exceeded the conventional significance threshold of 0.05. This result suggested that the difference in 28-day mortality between the two groups was not statistically significant.

**Table 3.2 McNemar test（*Γ* = 1.0）**

| *Γ* value | lower-bound *P* value | upper-bound *P* value |
| --- | --- | --- |
| 1.0 | 0.0462 | 0.0462 |
| 1.1 | 0.0201 | 0.0945 * |
| 1.3 | 0.0084 | 0.1641 * |
| 1.4 | 0.0035 | 0.2514 * |
| 1.5 | 0.0014 | 0.3497 * |
| 1.6 | 0.0006 | 0.4511 * |
| 1.7 | 0.0002 | 0.5486 * |
| 1.8 | 0.0001 | 0.6374 * |
| 1.9 | 0.0000 | 0.7145 * |
| 2.0 | 0.0000 | 0.7790 * |

**Table 3.3 The results of Rosenbaum Bounds sensitivity analysis of sepsis.**

| *Γ* value | *P* value |
| --- | --- |
| Cutoff *Γ* value | 1.1 |
| 1.0 | 0.155 |
| 1.1 | 0.0945(upper-bound P value) |

*Γ* = 1.0 indicates no unmeasured confounding, while *Γ* > 1.0 corresponds to increasing levels of hidden confounding. If the upper-bound p-value exceeds the significance level (α = 0.05), unmeasured confounding at that Γ level could eliminate statistical significance. A larger critical Γ value reflects greater robustness of the analytical results. * p > 0
